# Chlorophyll fluorescence analysis in diverse rice varieties reveals the positive correlation between the seedlings salt tolerance and photosynthetic efficiency

**DOI:** 10.1186/s12870-019-1983-8

**Published:** 2019-09-13

**Authors:** Yu-Chang Tsai, Kuan-Chuan Chen, Tung-Shan Cheng, Chuan Lee, Shih-Hung Lin, Chih-Wei Tung

**Affiliations:** Department of Agronomy, National Taiwan University, No. 1, Sec. 4, Roosevelt Rd, Taipei, 10617 Taiwan

**Keywords:** *Oryza sativa*, Photosynthetic efficiency, Chlorophyll fluorescence, Salt stress, Genetic variation, Subspecies, Genome-wide association

## Abstract

**Background:**

Photosynthetic efficiency might be a key factor determining plant resistance to abiotic stresses. Plants can sense when growing conditions are not favorable and trigger an internal response at an early stage before showing external symptoms. When a high amount of salt enters the plant cell, the membrane system and function of thylakoids in chloroplasts could be destroyed and affect photosynthetic performance if the salt concentration is not regulated to optimal values. *Oryza* species have salt-tolerant and salt-sensitive genotypes; however, very few studies have investigated the genetic architecture responsible for photosynthetic efficiency under salinity stress in cultivated rice.

**Results:**

We used an imaging-based chlorophyll fluorometer to monitor eight rice varieties that showed different salt tolerance levels for four consecutive days under control and salt conditions. An analysis of the changes in chlorophyll fluorescence parameters clearly showed the maximum quantum efficiency of PSII in sensitive varieties was significantly reduced after NaCl treatment when compared to tolerant varieties. A panel of 232 diverse rice accessions was then analyzed for chlorophyll fluorescence under salt conditions, the results showed that chlorophyll fluorescence parameters such as F_0_ and NPQ were higher in *Japonica* subspecies, ΦPSII of *Indica* varieties was higher than that in other subgroups, which suggested that the variation in photosynthetic efficiency was extensively regulated under salt treatment in diverse cultivated rice. Two significant regions on chromosome 5 were identified to associate with the fraction of open PSII centers (qL) and the minimum chlorophyll fluorescence (F_0_). These regions harbored genes related to senescence, chloroplast biogenesis and response to salt stress are of interest for future functional characterization to determine their roles in regulating photosynthesis.

**Conclusions:**

Rice plant is very sensitive to salinity stress, especially at young seedling stage. Our work identified the distribution pattern of chlorophyll fluorescence parameters in seedlings leaf and their correlations with salt tolerance level in a diverse gene pool. We also revealed the complexity of the genetic architecture regulating rice seedling photosynthetic performance under salinity stress, the germplasm analyzed in this study and the associated genetic information could be utilized in rice breeding program.

**Electronic supplementary material:**

The online version of this article (10.1186/s12870-019-1983-8) contains supplementary material, which is available to authorized users.

## Background

Photosynthesis is an essential process that generate energy to support plant growth. Components participating in photosynthetic machinery, such as photosynthetic pigments, photosystems, electron transport systems, gas-exchange processes and enzymes involved in carbon metabolism, are important for photosynthetic efficiency and could be potentially affected by abiotic stresses [1].

In recent years, technology to detect chlorophyll fluorescence in leaves has advanced rapidly, and its power to reflect photosynthetic efficiency in vivo needs to be examined. Light energy captured by chlorophyll molecules is used in photochemical reactions to drive photosynthesis or, if in excess, dissipated as heat (nonphotochemical quenching, NPQ) or emitted as chlorophyll fluorescence [2]. These three processes mutually compete with each other, e.g., increasing photosynthesis will lead to a decrease in extra energy dissipation; therefore, by measuring the yield of chlorophyll fluorescence, the efficiency of the photochemical reactions and the degree of heat dissipation could also be estimated.

When a high amount of salt was accumulated in the plant cell for a period of time, the membrane permeability and function of thylakoids in chloroplasts was damaged [3]; a gradual decrease in the activity of photosystems (PSI and PSII) and chlorophyll fluorescence was also observed [4]. Because the repair of photosystem II (PSII) was affected in salt-treated plants, photoinhibition was enhanced, and the photosynthetic efficiency was reduced [5]. The correlation between chlorophyll fluorescence and chloroplast ultrastructure, such as the morphology of thylakoids, under salt stress has been examined in barley and rice [4, 6, 7]. These studies showed that salt-tolerant varieties had reduced thylakoid swelling, relatively high PSII electron transport activity, slowly decreasing maximum chlorophyll fluorescence yield and delayed senescence.

Compared to its physiological and biochemical regulation, the genetic nature of photosynthetic efficiency has been less explored [8, 9]. The activity of plant photosynthesis could be expressed and quantified in many forms, such as rate of CO_2_ fixation (gas-exchange) per unit leaf area, production of carbohydrates or dry mass per plant, and chlorophyll fluorescence. To investigate the genetic characteristics of plant photosynthetic efficiency, researchers analyzed the properties of diverse genotypes: the response of photosynthetic light use efficiency (ΦPSII) to different light conditions in Arabidopsis [10], chlorophyll fluorescence kinetics under heat stress in wheat [11], leaf chlorophyll content in rice [12–14], photosynthetic responses of rice seedlings to salinity stress [15], cold responses of photosynthesis in Arabidopsis and maize [16, 17] and other traits (summarized in Table 1 of Flood et al. [8]). These results highlighted that photosynthetic efficiency was regulated differentially in diverse genetic backgrounds.

**Table 1:** The eight accessions used in the pilot experiment

ID	Name	Country of origin	Subspecies	Injury score^b^	Salinity tolerance level^c^
8777	Munao PS405	Philippines	Indica	7.86	S
IR28	IR28	Philippines	Indica	5.2	M to S
IR64	IR64	Philippines	Indica	5.12	M to S
223	Priano Guaira	Brazil	Japonica	5	M
Nona Bokra	Nona Bokra	India	Indica	2.1	M to T
149	Sinaguing	Philippines	Japonica	2	M to T
245^a^	Sab Ini	Egypt	Japonica	1.57	M to T
IR66946	FL478	Philippines	Indica	1	T

^a^Variety 245 was excluded from discussion due to its erratic behavior; it is likely that the tested samples were contaminated

^b^The injury score was the average of three replicates

^c^Salinity tolerance level was assigned to five classes based on the injury score. *M* Moderate (Injury score = 5), *S* Sensitive (Injury score > = 7), *T* Tolerant (Injury score = 1)

Several studies have examined changes in chlorophyll fluorescence parameters under salinity stress in rice. F_0_ (minimum fluorescence yield in the absence of photosynthetic light) increased under the salinity stress [7, 18, 19]. Chloroplast ultrastructure, such as the thylakoid membrane, was damaged by salt stress [20]. It is possible that increased F_0_ values were due to the disassociation of light-harvesting complex II (LHC II) and the PSII reaction center on swollen thylakoids [7]; however, F_m_ (maximum fluorescence yield in the absence of photosynthetic light) was decreased in plants under salinity stress compared with control plants [7, 18]. F_v_/F_m_ (maximum efficiency of PSII) is highly consistent (approximately 0.78–0.84) in different species under normal growth conditions; it was decreased in sensitive varieties under moderate salt stress or a long period of salt treatment [7, 15, 18, 19, 21, 22]. ΦPSII, which is also the effective PSII quantum yield, represents the fraction of absorbed energy used in photochemistry, which determines the efficiency of PSII. It is affected by the rate of electron transport or the concentrations of electron acceptors, e.g., NADP^+^, available at the acceptor side of PSI. The value of ΦPSII declined under moderate salt stress, and the tolerant lines exhibited higher values than the sensitive lines [15]. However, another study [23] had the opposite result; ΦPSII did not change, but the electron transport rate (ETR) declined, and they speculated that photosynthetically active radiation (PAR) was not uniform in the experiment. Another study also indicated that ΦPSII decreased severely when F_v_/F_m_ decreased slightly under different levels of salinity stress [7]. The parameter qL is the coefficient of photochemical quenching based on a lake model in which the PSII reaction centers are connected by shared antenna [24]. qL reflects the fraction of open PSII reaction centers, which is highly correlated with ΦPSII and declines under salt stress in sensitive varieties [21]. NPQ reflects the level of excess energy dissipation as heat. A previous study showed that the NPQ value increased in tolerant varieties and decreased in sensitive varieties under salt stress [21], but in another study, NPQ in both tolerant and sensitive lines was increased substantially under salt stress [23]. It is still not clear how NPQ is related to plant salt tolerance levels.

An image-based phenotyping platform has been applied to increase the accuracy and throughput of the trait evaluation process in studying the effect of abiotic stress in various crops [25, 26]. A recent study combined digital imaging and an automated robotic system (the Plant Accelerator) to measure the relative growth rate (RGR), transpiration rate (TR) and transpiration use efficiency (TUE) in diverse rice accessions and identified several new genetic loci controlling the early responses to salinity through association studies [27]. In this work, we aimed to understand how natural genetic variation correlates with plant photosynthetic efficiency under salinity stress using a chlorophyll fluorometer. The leaf chlorophyll fluorescence of eight rice varieties that exhibited different levels of salt tolerance were monitored for four consecutive days under control and salinity conditions. The results were examined to determine how internal chlorophyll fluorescence parameters correspond to the symptoms of salt toxicity in older seedlings. Based on these findings, we further investigated a diverse panel of 232 rice cultivars for their seedling leaf chlorophyll fluorescence performance under salt conditions. The genetic architecture controlling photosynthetic efficiency was analyzed based on the association of selected chlorophyll fluorescence parameters and SNP variations.

## Results

### Evaluating the performance of leaf fluorescence parameters in time series under control and salinity conditions using eight rice varieties that show different salt tolerance levels

In the pilot experiment, we aimed to identify the optimal seedling stage and target leaf tissue displaying a wide range of chlorophyll fluorescence variation under salt stress. We selected eight rice varieties based on their salt tolerance level and classified them as tolerant, moderate and sensitive (Table 1).

**Fig. 1:**
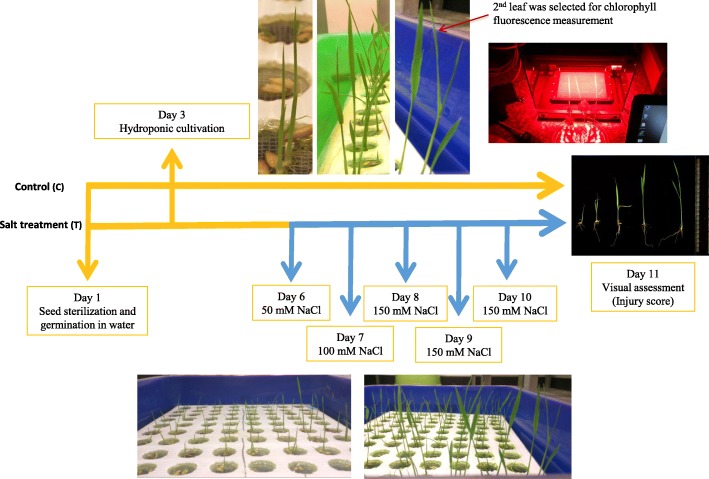
Experimental workflow of the rice salinity regime and phenotyping at the seedling stage. Rice seeds were sterilized and germinated at 37 °C for two days. Germinated seeds were transferred to the hydroponic system on day 3. Salinity treatment was started from 50 mM NaCl on day 6 and progressively increased to 150 mM on day 8, which allowed the seedling to acclimate to salt stress. The fully developed second leaf was selected for chlorophyll fluorescence measurement from day 8 to day 11. Salt tolerance level was evaluated and assigned an injury score on day 11

To synchronize seed germination, we soaked sterilized seeds in water at 37 °C for 2 days until the embryonic plumule protruded. After growing in the hydroponic system for 3 days, rice seedlings were treated with salt levels that increased daily (50, 100, 150 mM of NaCl) over subsequent days (days 6, 7, 8) and then maintained at 150 mM for 3 days for a visual evaluation of salt tolerance (injury score, IS) on day 11. As shown in Fig. 1, the second leaf was the major photosynthetic organ from day 6 to day 9 and was fully expanded on day 8 under both control and salt conditions. To assess the changes in the fluorescence parameters under the control and salt treatments at the young seedling stage, the second leaves of eight varieties were evaluated before starting 150 mM NaCl treatment (day 8) and 24, 48, and 72 h after 150 mM NaCl treatment (day 9, 10, 11) using a pulse-amplitude modulation (PAM) fluorometer (Fig. 1). The second leaf of the control group from the same eight varieties was evaluated at the same time point.

**Fig. 2:**
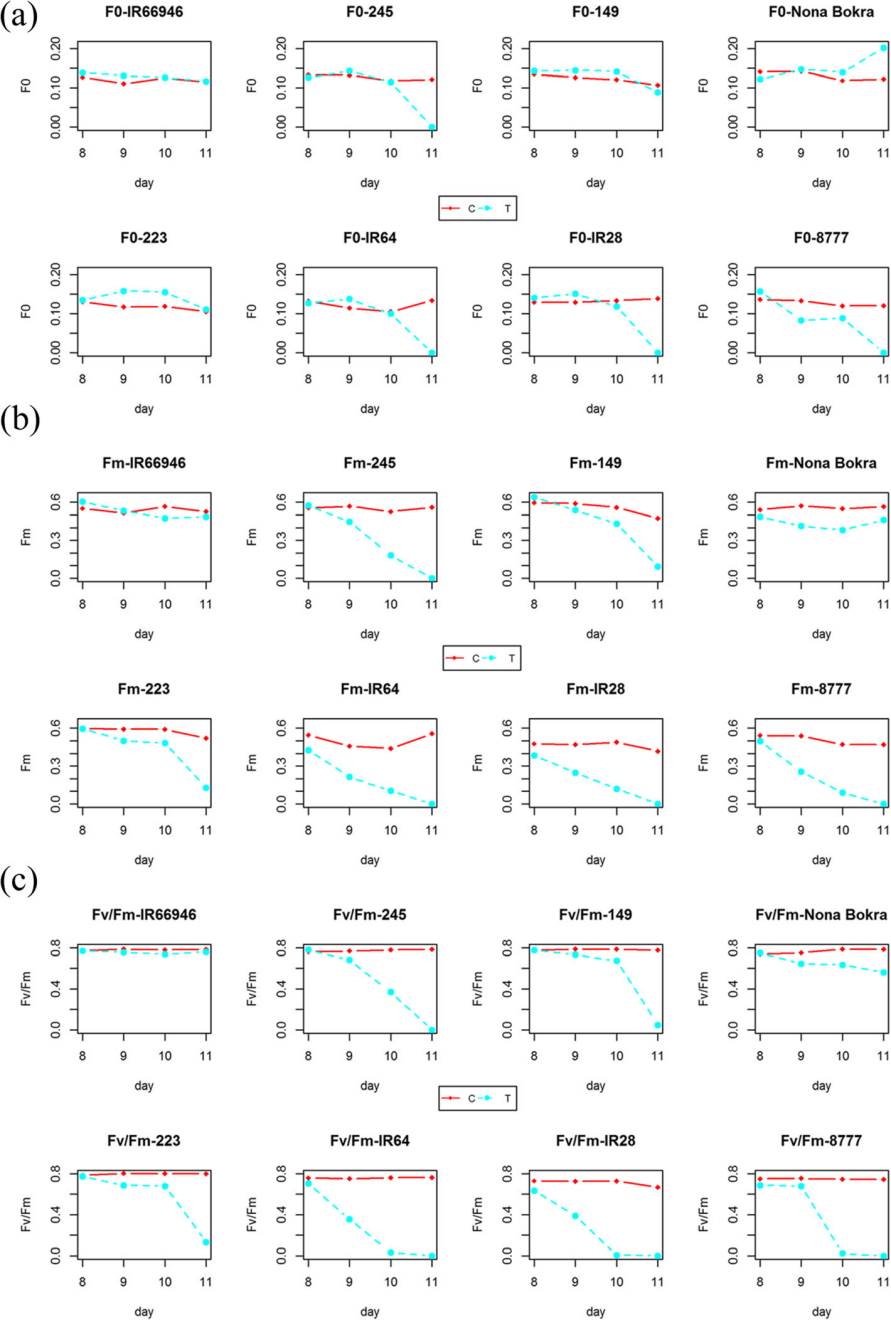
Daily changes in chlorophyll fluorescence parameters in eight rice varieties under control and salt conditions. The data in (**a**), (**b**), and (**c**) represent F_0_, F_m_ and F_v_/F_m,_ respectively. Each data point represents the average value of 3 replicates. Red line represents control sample; blue line represents salt treated sample

**Fig. 3:**
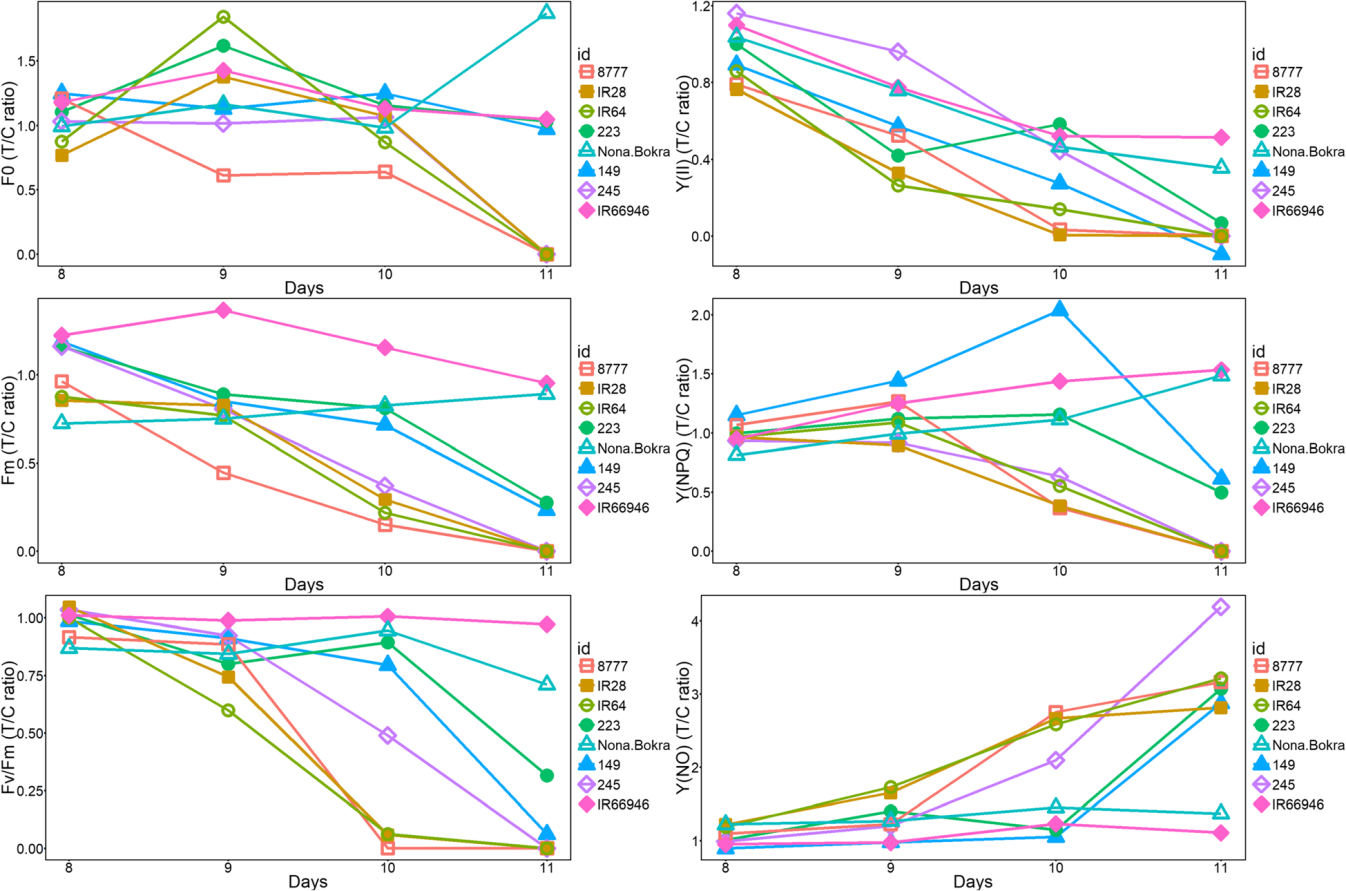
Regulation of the chlorophyll fluorescence index in eight rice varieties under salinity stress. The T/C ratio of chlorophyll fluorescence (F_0_, F_m_, and F_v_/F_m_) and quantum yield (Y(II), Y(NPQ), and Y(NO)) parameters were determined in control (C) and treatment (T) groups from day 8 to day 10. Each variety is indicated by a different colored line and symbol. 149: Blue/ filled triangle, 223: green/ filled circle, 245: violet/ open diamond, 8777: orange/ open square, IR28: brown/ filled square, IR64: light green/ open circle, IR66946: pink/ close diamond, Nona Bokra: jade/ open triangle

As shown in Fig. 2 and S1, the changes in six chlorophyll fluorescence parameters were relatively stable in the control group (red line) compared to the salt treatment group (blue line) from day 8 to day 11. A varietal difference was observed in the parameters of the control group such as ΦPSII, qL and NPQ, which strongly suggested that the photosynthetic potential varies in nature by genotype and could be regulated genetically. The F_0_, F_m_ and F_v_/F_m_ values of all varieties were stable under control conditions over the observation period (red line in Fig. 2) but declined at different rates in the tested accessions except the extremely tolerant variety IR66946 under salt treatment (blue line in Fig. 2). For the parameters associated with energy partitioning, such as ΦPSII, qL and NPQ, the pattern of chlorophyll fluorescence was difficult to interpret when control and salt treatment profiles were compared side by side across varieties (Additional file 7: Figure S1). It is likely that the capacity of the photosystem was already compromised in both the control and salt treatment groups, which compounded with the natural variation in photosynthetic efficiency that already existed between genotypes. To accurately evaluate the salt tolerance level through chlorophyll fluorescence across genotypes, the ratio of treatment to control (T/C) values for each parameter was then calculated and used to infer the regulation of photosynthetic efficiency (Fig. 3; Additional file 2: Table S2). Y(II) significantly declined in the second leaves of all seedlings treated with 150 mM NaCl compared to the control seedlings from days 8 to 11. Both the quantum yield of regulated [Y(NPQ)] and nonregulated [Y(NO)] energy dissipation were elevated from day 8 to day 9 in the sensitive varieties (Fig. 3). The Y(NPQ) increased from days 8 to 11 in the tolerant varieties IR66946 and Nona Bokra but decreased from day 9 to 11 in the sensitive varieties. The NPQ was regulated in response to salt stress in moderate varieties 149 and 223 and in tolerant varieties Nona Bokra and IR66946 at day 10. The actual quantum yield of nonregulated NPQ, Y(NO), remained low in the moderate and tolerant varieties (IR66946, Nona Bokra, 149 and 223) compared to the sensitive variety 8777 under salt treatment at day 10.

### The time point displaying the maximum difference in photosynthetic efficiency in seedling leaves of diverse varieties under salt stress

The second leaves displayed no significant visual difference between the control and salt-treated groups before the salt concentration increased to 150 mM NaCl on day 8. Twenty-four hours after 150 mM NaCl incubation began (day 9), the seedlings of the sensitive varieties 8777 and IR28 started to show weak senescence symptoms in the second leaf. Forty-eight hours after 150 mM NaCl incubation began (day 10), the moderate and sensitive varieties started withering; moderate to tolerant varieties started showing mild senescence symptoms on day 11, and the extremely tolerant varieties still looked healthy. We suspected that the photosynthetic machinery had already been affected since the first day of salt treatment (50 mM on day 6, Fig. 1); however, the phenotypic effect was not shown until the leaf tissue started senescing and dying from day 9 to day 11, which depended on the tolerance level of the variety. We closely examined the changes in the six chlorophyll fluorescence parameters across eight varieties from day 8 to day 11. The pattern of each parameter varied greatly among eight varieties in our time-series observation, suggesting that chlorophyll fluorescence is a sensitive and complex phenomenon that reflects the seedling’s salt tolerance level. F_v_/F_m_ and ΦPSII values of salt-treated moderate and sensitive varieties were close to 0 on day 10 (Fig. 2c and Additional file 7: Figure S1), which cannot be treated as a quantitative phenotype in a genome-wide association study (GWAS). We then decided to measure six chlorophyll fluorescence parameters on day 9 in 223 diverse rice accessions under salt stress and used these measurements as quantitative traits for follow-up association analysis (Additional file 3: Table S3).

### Distribution of chlorophyll fluorescence parameters under salinity stress in 232 diverse rice varieties

**Fig. 4:**
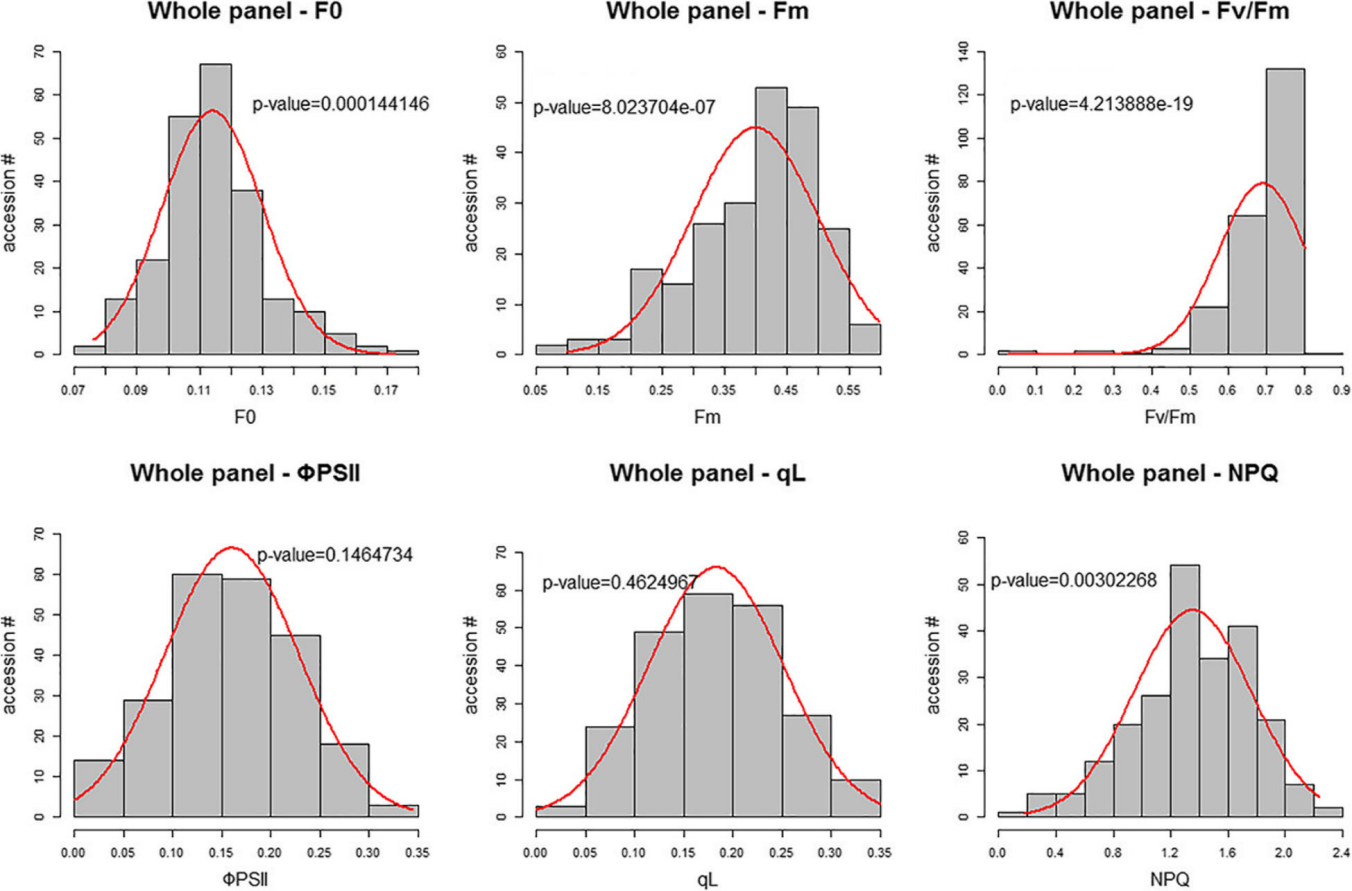
Distribution of six chlorophyll fluorescence parameters in 232 diverse varieties. Each histogram showed the distribution of chlorophyll fluorescence parameter in 232 rice accessions. The normality of each parameter was examined using the Shapiro-Wilks test. The *P*-value of each test is provided

After 150 mM salt treatment for 2 days, F_v_/F_m_ was between 0.7 and 0.8 in the majority of the accessions, as we expected; F_0_, F_m_, ΦPSII, qL and NPQ values showed a normal distribution in 232 diverse accessions (Fig. 4). We then investigated the variation in the parameters in five subgroups and two subspecies and found that *Japonica* subspecies have significantly higher F_0_ and NPQ values than *Indica* subspecies (*P*-value < 0.0001 by Student’s *t*-test, Additional file 8: Figure S2); within *Japonica* subspecies, *tropical japonica* varieties have higher F_0_ values than other varieties, and *aromatic* varieties have higher NPQ values (Additional file 8: Figure S2a and f). ΦPSII of the *Indica* subgroup is slightly higher than that in the other four subgroups (Additional file 8: Figure S2d), and for F_m_, F_v_/F_m_ and qL, we did not detect a significant difference between the two subspecies (Additional file 8: Figure S2b, c and e). The value of chlorophyll fluorescence parameters were widely distributed in each subgroup, which could reflect the extensive genetic variation in photosynthetic efficiency in rice.

**Fig. 5:**
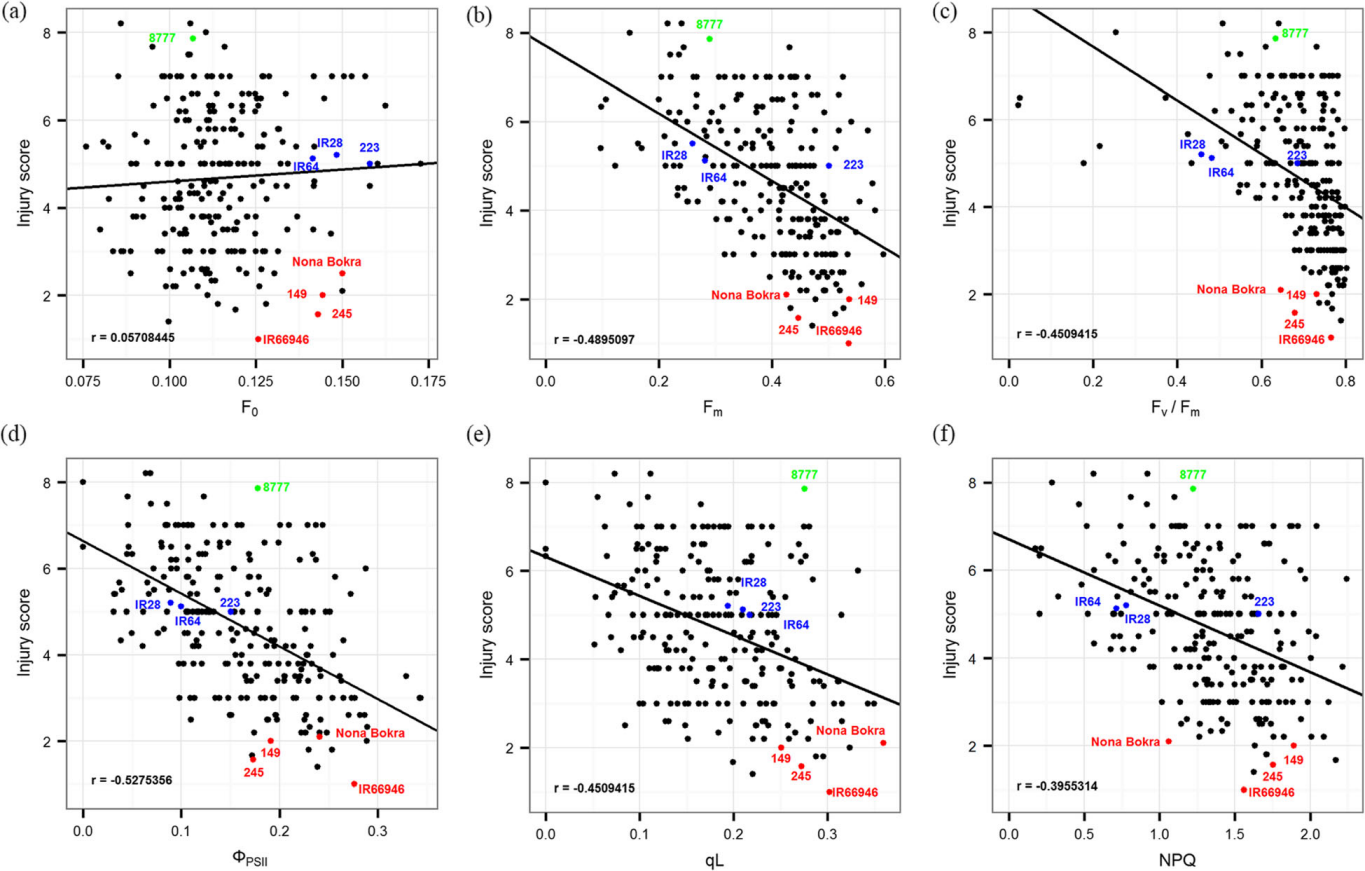
Pattern of correlation between injury score and each chlorophyll parameter. **a** to **f** showed distribution of injury score and F_0_, F_m_, F_v_/F_m_, ΦPSII, qL and NPQ, respectively. Pearson correlation coefficient (r) represents the level of correlation between two traits. The variety labeled in color corresponds to the salt tolerance level described in Table 1

**Fig. 6:**
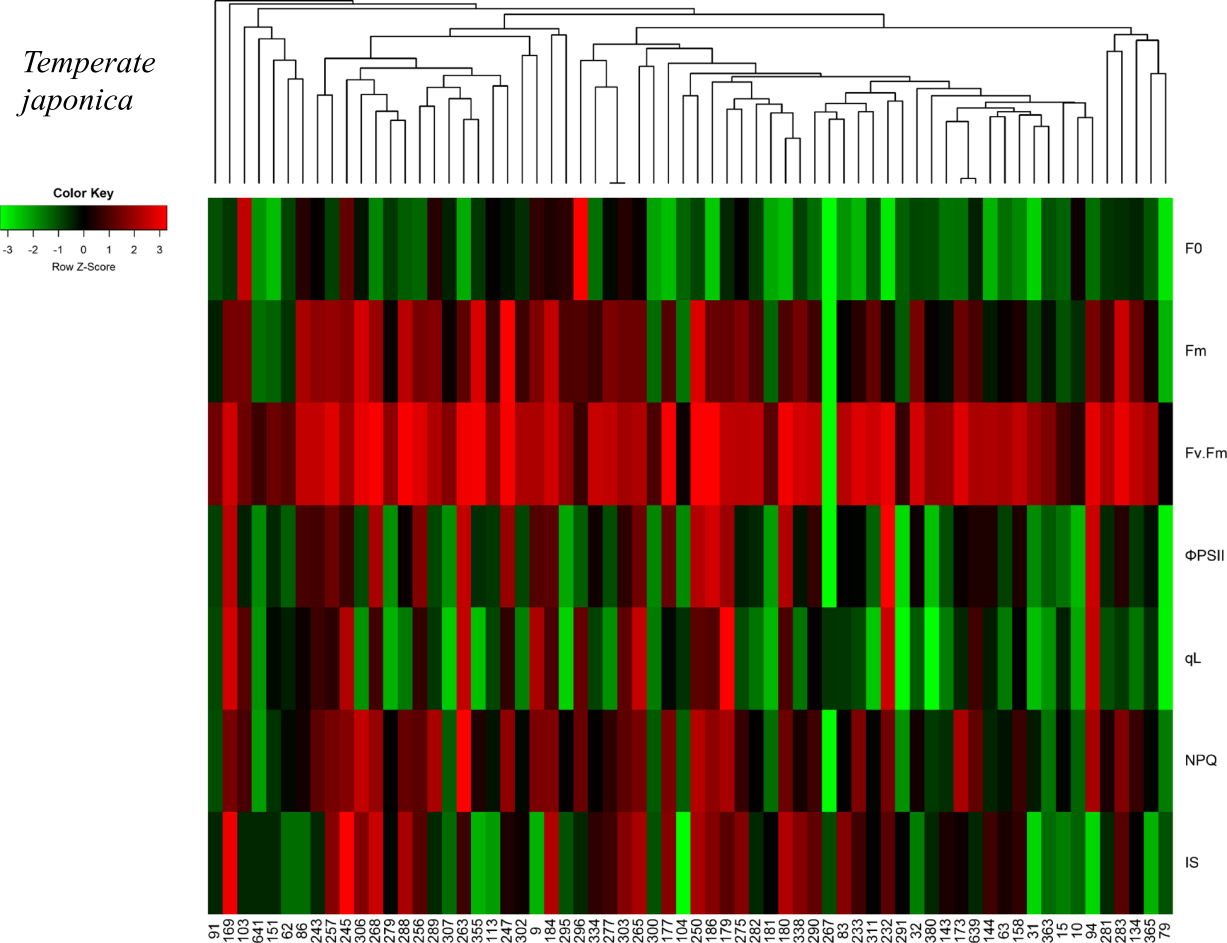
Genetic relationships and phenotypic distributions of varieties in *temperate japonica* populations. Each column represents a single variety, and the sample ID is labeled at the bottom of the heatmap. Each row represents the chlorophyll fluorescence parameter and injury score of each variety. The colors closest to red indicate that the value of each chlorophyll fluorescence parameter is higher than its average value of all accessions; for injury score, the red colors represent the lower injury scores, which suggest that the variety is more tolerant to salt toxicity. The color key of the Z-score was calculated from the distance between the raw score and the population mean in units of the standard deviation

To evaluate how the chlorophyll fluorescence of these 232 rice accessions recorded on day 9 was related to their visual salt toxicity symptoms (i.e., injury score) observed on day 11, we plotted these variables in pairs and calculated their correlations (Fig. 5 and Additional file 4: Table S4). The five fluorescence parameters other than F_0_ were moderately correlated with injury score. Considering that chlorophyll fluorescence parameters differed among subpopulations, we investigated whether these parameters and the salt tolerance level were correlated by genetic relatedness in each subpopulation (*temperate japonica* in Fig. 6 and other five subpopulations in Additional file 9: Figure S3). Colors in the heatmap near red indicated that the value of each chlorophyll fluorescence parameter was higher than the average value of all accessions. For the injury score, red represented a lower injury score, which suggested that the variety was more tolerant to salinity toxicity than the other varieties. These results clearly showed that rice accessions with related genetic backgrounds do not necessarily share similar chlorophyll fluorescence patterns, which was reflected by their diverse responses to salt toxicity, strongly supporting the complexity of salt response mechanisms.

### GWAS of chlorophyll fluorescence parameters under salt stress

**Fig. 7:**
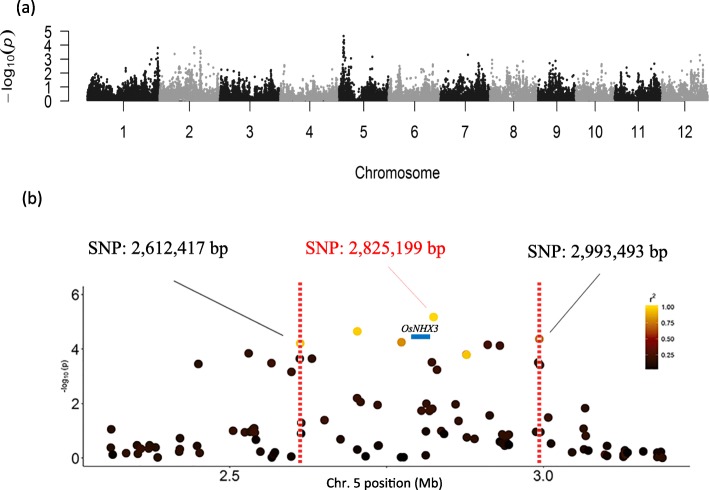
GWAS of F_0_ in 232 diverse varieties and LD pattern of the most significant SNP. **a** Manhattan plot showing the significance of each SNP tested by a mixed linear model. **b** The most significant SNP is at 2,825,199 bp. The dot color of each SNP represents its LD with the most significant SNP, and the decay of the LD is bordered by two vertical dashed lines. The location of the *OsNHX3* gene is marked with a blue bar

To identify the chromosomal regions that are potentially involved in the photosynthetic efficiency response to salt stress, we conducted GWAS using five fluorescence parameters in three panels – a whole panel (232 accessions, Fig. 2), a *Japonica*-specific panel (165 accessions, Additional file 10: Figure S4) and an *Indica*-specific panel (66 accessions, Additional file 11: Figure S5). F_v_/F_m_ was excluded because of its narrow variation and skewed distribution (Fig. 2). For the whole panel, the P model, K model and P + K model were applied to reduce false-positive signals compared to the naïve model results; for *Japonica*-specific panels, only the K model was applied. According to the quantile-quantile (QQ) plot, the significant SNPs were selected by a *P*-value < 10^− 4^ in the P model and a *P*-value < 10^− 3^ in the K and P + K models, and the singleton SNP was not considered in further analysis. Manhattan plots and QQ plots of the SNPs associated with each chlorophyll fluorescence parameter in the whole panel and two subspecies-specific panels are provided in supplementary files (Additional file 12: Figure S6). To identify candidate genes in significant genomic regions, we calculated the linkage disequilibrium (LD) between the most significant SNP and its neighboring SNPs. The chromosomal region in high LD with a significant SNP could potentially harbor the causal gene affecting the photosynthetic efficiency under salt stress. Several photosynthesis-associated loci under salt stress were identified in this study (Table 2). Significant peaks on chromosome 1 (41.7–42.5 Mb) were detected for five different parameters (F_0_, F_m_, ΦPSII, qL, NPQ). This region overlapped with one quantitative trait locus (QTL), and this QTL was related to seedling height under salt stress [28]. The *Ygl8* gene encoding a UMP kinase (LOC_Os01g73450) was found near this significant SNP cluster. UMP kinase may control the UMP/UMP/UTP level in chloroplasts. Chlorophyll biogenesis was affected in a mutant of *Ygl8*, and the chloroplast ultrastructure of *Ygl8* showed that the lamellae were intricately stacked and swollen [32]. Another significant peak, located on chromosome 6 from 2.9 to 3.05 Mb, was observed for four traits (F_m_, ΦPSII, qL, NPQ) (Table 2). F_m_ and NPQ are related to the dissipation of excess energy as heat, and significant SNP peaks were detected in three chromosome regions (Table 2). Here, we used F_0_ and qL as examples to describe the gene mining process in detail. The most significant SNP peak associated with F_0_ in the whole panel and *Japonica*-specific panel was on chromosome 5 (Fig. 7a). We investigated the LD structure flanking the most significant SNP at 2,825,199 bp, and the region between 2,612,417 and 2,993,493 bp was identified based on the decay of the LD (Fig. 7b). According to the Plant Genes 60 database in Gramene Mart (www.gramene.org), 61 genes were annotated in this area (Additional file 5: Table S5). *OsNHX3* (*LOC_Os05g05590* or *Os05g0148600*, located at chr5: 2,777,359 - 2,783,546 bp) encoded the NHX-type Na^+^/H^+^ antiporter and was near the most significant SNP (Fig. 7b). A previous study showed that the expression of *OsNHX3* was upregulated by salt treatment [35]. Another gene (*LOC_Os05g05600* or *Os05g0148700*) next to *OsNHX3* was called the “senescence-associated protein OSA15” and is located in the chloroplast, but how it regulates leaf senescence in rice needs to be examined. Two other genes (*LOC_Os05g05830* or *Os05g0150800* and *LOC_Os05g05950* or *Os05g0151400*) were also predicted to target chloroplasts, but their roles in photosynthetic efficiency need to be confirmed. For qL, a total of seven significant SNP peaks (*P*-value < 10^− 3^ based on the P + K model) located among chromosomes 1, 2, 5, 6 and 12 were detected in the whole panel (Additional file 13: Figure S7a). The LD around the most significant SNP (at 26,473,167 bp) on chromosome 5 was analyzed (Additional file 13: Figure S7b). Based on the decay of the LD, the region harboring candidate genes was between 26,164,849 and 26,862,431 bp, and 113 genes were annotated in this region (Additional file 6: Table S6). Four QTLs related to root length and root dry weight under salt stress [30] and salt tolerance level [33] resided in this region (Table 2). The gene *OsCBL4* (*OsSOS3*, *LOC_Os05g45810* or *Os05g0534400*) encoded an EF-hand-type calcineurin B-like protein in this region (Additional file 13: Figure S7b). OsCBL4 has the same function as AtSOS3 in Arabidopsis and is involved in the regulatory pathway controlling intracellular Na^+^ and K^+^ homeostasis and salt tolerance [36].

**Table 2:** The significant SNPs detected in a GWAS for five chlorophyll parameters and associated QTLs and genes

Trait	SNP ID	Chr.	Position (IRGSP-1.0)	Statistical model	*P-*value	Known QTL	Known gene	
NPQ	id1003509	1	4,209,827	All - P	4.17E-07	QKr1.1 [28]; qRNC-1, qRRNC4, qRKC1, qRRKC1, qRNa/K1–1, qRRNa/K1–1 [29]		
F0	id1004960	1	6,508,269	IND - K	2.17E-04	qRL-1, qNAUP-1b [30]		
qL	id1016791	1	28,594,265	All - P + K	7.95E-05	qNAUP-1a, qFWSH-1 [30]; QKr1.2, Qnar/Kr1 [28]		
	id1016825	1	28,599,615	All - K	8.10E-04			
NPQ	id1024223	1	38,162,791	All - P	4.95E-05			
ΦPSII	id1026852	1	41,787,616	All - P	3.99E-05	qSH1–3 [31]	Ygl8 (LOC_Os01g73450, Zhu et al. [32])	
	id1027309	1	42,134,803	All - K	2.20E-04			
NPQ	id1026852	1	41,787,616	All - P	4.67E-06	qSH1–3 [31]		
F0	id1027269	1	42,082,183	All - K	1.01E-04	qSH1–3 [31]		
				JPN - K	7.39E-04			
	id1027563	1	42,339,408	All - P	7.63E-06	qSH1–3 [31]		
				All - P + K	1.53E-04			
qL	id1027309	1	42,134,803	All - P	4.26E-05	qSH1–3 [31]		
				All - K	5.44E-04			
Fm	id1027821	1	42,581,986	All - P	1.55E-05	qSH1–3 [31]		
ΦPSII	id2001842	2	3,253,759	All - P	7.78E-05			
				All - K	3.05E-04			
				All - P + K	2.35E-04			
qL	id2001842	2	3,253,759	All - K	7.90E-04	qSST2 [33]		
NPQ	id2006687	2	16,648,368	All - P	4.10E-05	qPL-2 [30]; QSsst2b, QDss2b [34]		
NPQ	id2007192	2	17,927,911	All - P	5.81E-05	qPL-2 [30]; QSsst2b, QDss2b [34]		
F0	id2008158	2	20,817,846	All - P	7.91E-05	QDss2a, QSkc2, QKna2 [34]		
				All - K	2.06E-04			
				All - P + K	1.41E-04			
NPQ	id2008679	2	21,812,170	All - P	5.36E-05	QDss2a, QSkc2, QKna2 [34]		
F0	id2010410	2	24,565,561	All - P + K	2.62E-04	QDss2a, QSkc2, QKna2 [34]; qRRNa/K1–2 [29]		
	id2010572	2	24,696,197	All - P	1.25E-05			
	id2010581	2	24,713,963	All - K	5.03E-04			
NPQ	id2010932	2	25,236,351	All - P	5.50E-05	QDss2a, QSkc2, QKna2 [34]; qRRNa/K1–2 [29]		
Fm	id4000429	4	686,361	All - P	1.90E-05			
				All - K	7.32E-04			
qL	id4002349	4	5,526,924	IND - K	3.61E-04	qRL4 [30]		
Fm	id4002319	4	5,487,935	IND - K	5.13E-04			
F0	id5001540	5	2,773,921	All - P	1.70E-06	QKs5 [28]	OsNHX3 (LOC_Os05g05610, Fukuda et al. [35])	
	id5001584	5	2,825,199	All - K	9.66E-05			
				All - P + K	6.82E-06			
				JPN - K	3.06E-04			
Fm	id5001738	5	3,064,389	IND - K	2.25E-05	QSst5, QDss5a, QDss5b [34]		
NPQ				IND - K	7.46E-04			
qL	id5012588	5	26,473,167	All - P	3.40E-06	qRL-5, qDWRO-5a, qDWRO-5b [30]; qSST5 [33]	OsCBL4 (LOC_Os05g45810, Martinez-Atienza et al. [36])	
				All - K	5.61E-04			
				All - P + K	4.95E-04			
ΦPSII	id6002261	6	2,930,916	All - P	8.97E-07			
				All - P + K	4.12E-05			
	id6002494	6	3,113,730	All - K	6.48E-05			
				IND - K	8.00E-04			
Fm	id6002261	6	2,930,916	All - P	2.77E-05			
				All - K	5.39E-04			
				All - P + K	4.79E-04			
qL	id6002261	6	2,930,916	All - P	3.90E-05			
				All - P + K	5.85E-04			
	id6002286	6	2,974,286	All - K	3.95E-04			
NPQ	id6002389	6	3,042,568	All - P	2.10E-06			
				All - P + K	4.59E-04			
NPQ	id6005326	6	8,187,911	All - P	1.31E-08	qDRW6 [31]		
				All - K	3.98E-04			
				All - P + K	2.35E-04			
NPQ	id6006336	6	10,184,310	All - P	3.32E-05	qDRW6 [31]		
				All - K	1.70E-04			
				All - P + K	1.83E-04			
				JPN - K	2.29E-04			
NPQ	id7000336	7	1,865,306	All - P	3.43E-05			
F0	id7003938	7	22,819,640	All - P	2.00E-05	QSkc7; [34] qSST7 [33]		
qL	id10000081	10	407,964	JPN - K	6.21E-04			
ΦPSII	id10003594	10	13,662,830	All - K	5.63E-04			
				All - P + K	4.98E-04			
				JPN - K	3.21E-04			
Fm	id10003623	10	13,782,039	All - P	3.03E-05			
Fm	id10006610	10	20,943,116	All - P	1.43E-06			
NPQ	id11008520	11	22,516,795	All - P	6.39E-06	qSNC11 [37]		
Fm	id11008950	11	23,178,265	All - P	4.04E-06	qSNC11 [37]		
Fm	id12001261	12	3,059,845	All - K	4.65E-05			
				All - P	1.30E-06			
				JPN - K	5.35E-05			
	id12001332	12	3,223,671	All - P + K	6.34E-05			
NPQ	id12001256	12	3,027,442	All - P	1.23E-05			
qL	id12001769	12	4,044,980	All - P + K	6.57E-04	qRRNC12 [29]		
ΦPSII	id12004053	12	10,470,139	All - P	5.14E-06	qRRNC12 [29]; qSUR12, qSES12, qCNL12 [38]		
				All - K	3.54E-04			
ΦPSII	id12007270	12	21,906,089	All - P	1.48E-04	QSst12, QDss12 [34]; qSUR12, qSES12, qCNL12 [38]		
ΦPSII	id12008292	12	23,734,278	All - P	2.45E-05	qSH12.1, qSH12.2, qDSW12.1 [31]; qSUR12, qSES12, qCNL12 [38]		
				All - K	4.67E-04			
qL	id12008234	12	23,663,599	All - P	5.21E-05	qSH12.1, qSH12.2, qDSW12.1 [31]; qSUR12, qSES12, qCNL12 [38]		
				All - K	8.73E-04			
ΦPSII	id12010084	12	27,378,310	All - P	1.65E-05	qSUR12, qSES12, qCNL12 [38]		
				All - K	1.76E-04

## Discussion

To explore the potential genetic resources for salinity-tolerant breeding in rice, chlorophyll fluorescence parameters could serve as reliable selection indicators, the pixelated chlorophyll fluorescence data can be used to evaluate the level of stress tolerance days before the injury symptom were expressed externally. The chlorophyll fluorescence parameters F_0_ and F_m_ were directly determined from the leaves, however, the alteration in F_0_ can be attributed to PSI fluorescence, which can be positively or negatively affected under stress [39]. In our study, F_0_ had the lowest correlation with the injury score in both the pilot experiment and the 232 varieties experiment. Among the chlorophyll fluorescence parameters, F_m_ had a higher correlation with the injury score in our pilot experiment than the others. However, ΦPSII had the highest correlation of the parameters with the injury score in 232 diverse varieties. Considering that the whole panel includes *Indica*- and *Japonica*-specific panels, the correlation of injury score and the six chlorophyll fluorescence parameters was better in each subgroup than in the whole. In the *Indica*-specific panel, the correlation of the injury score with F_m_ was improved from − 0.490 to − 0.617, which was also similar to the value in the pilot experiment that included five *Indica* rice varieties. In addition, the correlations of NPQ and ΦPSII to the injury score were also improved in the subgroups. The correlation of injury score with ΦPSII was slightly better when in the *Japonica* varieties alone. The pattern of chlorophyll fluorescence parameters and how they correlated with injury score may indicate the different strategies operating in *Indica* and *Japonica* rice for survival of salinity stress.

The decrease in F_m_ was shown to be an indicator of a PSII reaction center made inactive by the stress [40]. In addition, the increase in F_0_ can also be a negative trait associated with stress, which could result in the separation of LHCII and PSII or the inactivation of the PSII reaction center [41, 42]. In our pilot experiment, F_0_ increased for all varieties except the most sensitive variety “8777” from day 8 to day 9 under salt stress and then gradually declined until the end of the 150 mM salt treatment in most varieties (not the two extremely tolerant varieties Nona Bokra and IR66946). It is possible that the changes of F_0_ and F_m_ were due to the damages of the chloroplast, which could be seen in the ultrastructure of the membrane system [7, 18, 20]. In addition, the malfunction of proteins involves in photosystem II or I could also relate to the reduction of F_m_ [7]. Unlike the tolerant rice varieties, which have stable F_v_/F_m_ and F_m_ values, sensitive varieties exhibited significantly decreased F_0_ and F_m_ values after day 9, which may indicate severe damage to the antenna structure or inactivation of the PSII reaction center. We suspected that the chloroplast ultrastructure of extremely sensitive variety 8777 was already severely damaged, since the seedling was already grown in 50–100 mM saline solution for 2 days (from day 6 to day 7). We anticipate that the membrane system of variety 8777 was relatively fragile compared to that of other sensitive varieties tested in our study, which was also reflected by the low F_v_/F_m_ ratio of variety 8777.

PSII efficiency can be determined by chlorophyll fluorescence indexes, which include Y(II), Y(NPQ), and Y(NO). These indexes represent the total energy distribution in the photosynthetic reaction center in PSII. Reduction in Y(II) levels and increased Y(NPQ) and Y(NO) levels have been shown to be responses to salt stress in cucumber [43]. In our study, the dynamics of Y(II), Y(NPQ) and Y(NO) from day 9 to day 11 in the tolerant lines (IR66946, Nona Bokra) and sensitive lines (8777, IR28) strongly suggest that enhancing PSII efficiency could improve the salt tolerance; therefore, the quantum yield of energy dissipation in PSII (including Y(NPQ) and Y(NO) components) may provide a hint of the different salinity tolerance levels among diverse rice varieties. The increase in Y(NPQ) depends on the formation of a transthylakoid pH gradient, and proton pumping appears to be inefficient in the sensitive lines [44]. Low Y(NPQ) values may also represent low xanthophyll cycle activity [45, 46], while Y(NPQ) is an indicator of ΔpH-dependent dissipation of excess light energy in the PSII antennae that causes photoinhibition or the xanthophyll cycle [47–49]. Y(NO) corresponds to nonregulated dissipation of excess energy and may be used as a stress indicator [50]. Combining responses to salinity and submergence stresses, ETR, qN, qP and Y(NO) can be used as indicators to distinguish the stress tolerances of different rice genotypes [51].

Associating photosynthetic efficiency with the level of salinity tolerance is challenging, especially when photosynthetic performance and plant growth rate vary among diverse genotypes regardless of salt treatment. We know that the accumulation of excess salt in the plant cell triggered internal physiological changes before external symptoms appeared [52]. In our study, visible senescence was not detected in the salt-treated varieties during day 6 to day 9, and the fluorescence parameters of the second leaf on day 9 were partially correlated with the injury score assessed on day 11 in 232 diverse varieties. Therefore, we hypothesized that these results could be due to the existence of multiple salt tolerance mechanisms in the diverse germplasms. Three major salt tolerance mechanisms were proposed: tissue tolerance, osmotic tolerance and ion exclusion [53]. Considering that all accessions received the same amount of salt treatment from day 6, the varieties with better tissue tolerance would mobilize the sodium to vacuole more efficiently and leave the chloroplast with only minor damage to conduct photosynthesis. If the varieties mainly generate salt tolerance through ion exclusion from roots, the chlorophyll fluorescence detected in the leaf may mostly reflect the natural aging (senescence) process in plants and not the net toxic effect from salt accumulation. As described in Negrão et al. [54], it is difficult to differentiate cause-effect relationships between photosynthesis and growth reduction when quantifying the effects of salinity on photosynthesis; therefore, the phenotypes associated with chlorophyll biosynthesis could confound the interpretation of association results. Through our experimental process, we identified several chromosomal regions associated with chlorophyll fluorescence parameter variation, some significant SNPs closely linked to the genes which were previously shown to be involved in salt tolerance (e.g. *Ygl8*, *OsNHX3, OsCBL4*), however, the effects of most novel SNPs were small (R^2^ less than 10%) and could be partially attributed to developmental aging variation in the tested varieties. A careful experimental design that considers the natural senescence process is required when the objective is to identify the chromosomal regions that are responsive solely to salinity stress.

## Conclusions

Is photosynthetic efficiency a quantitative trait and how does it vary under salinity stress? In this study, we tried to answer these two questions by phenotyping a diverse panel of rice seedling chlorophyll fluorescence under salt stress. Our results revealed the quantitative trends in photosynthetic efficiency, and a significant genetic heterogeneity in photosynthetic efficiency that implied that photosynthetic efficiency is highly genetic background-dependent was detected. Considering that the population size is relatively small in our study, we recommend that future association studies should increase the number of varieties to enrich favorable alleles; alternatively, using a biparental mapping population created by crossing tolerant and susceptible varieties that show contrasting chlorophyll fluorescence imaging patterns would allow us to dissect and identify the genetic components and genes that control photosynthetic efficiency.

## Methods

### Plant material and growth conditions

Eight rice accessions, including four salt-tolerant, three moderate and one salt-sensitive accessions, were evaluated in the pilot experiment (Table 1). For the GWAS experiment, 232 accessions from 71 countries were used for the study (Additional file 3: Table S3). This global panel included 5 subpopulations: 59 *tropical japonica*, 65 *temperate japonica*, 38 *indica*, 21 *aus*, 9 *aromatic* and 42 admixed accessions. Rice accessions were obtained from the USDA-ARS, Dale Bumpers National Rice Research Center, Stuttgart, Arkansas, Genetic Stocks Oryza Collection (www.ars.usda.gov/GSOR). Each accession was genotyped on a 44,000 SNP array as described in Zhao et al. [55]. After removing the SNPs with missing rates larger than 10% and minor allele frequencies less than 5%, a total of 29,195, 14,750, and 21,514 SNP markers were identified in the 232, 165 and 66 accessions that belong to the whole panel, *Japonica*-specific panel, and *Indica*-specific panel, respectively.

Sterilized seeds were soaked at 37 °C in water for 2 days to synchronize the germination, and germinated seeds were placed in a hydroponic culture system in a phytotron on day 3. Three replicates for each rice accession were included in each experiment. The photoperiod was set to a 12-h-day/12-h-night cycle, and the temperature was set at 28 °C / 25 °C (day/night). The light intensity was controlled at 350 μmole m^− 2^ s^− 1^. The hydroponic solution was a half-strength Kimura B solution [56]. After growing in the hydroponic system for 3 days, rice seedlings were treated with salt levels that increased daily (50, 100, 150 mM) over subsequent days (days 6, 7, 8) and then maintained at 150 mM for 3 days for a visual evaluation of salt tolerance (injury score, IS) on day 11 (Fig. 1). In comparison, the control group was grown under the same conditions without any added salt. The hydroponic solution was refreshed every 3 days, and the pH of the culture solution was adjusted to 4.9.

In the pilot experiment, chlorophyll fluorescence imaging was conducted on days 8, 9, 10, and 11 in the control and treatment groups; for the GWAS experiment, chlorophyll fluorescence imaging was conducted on day 9 in the salt treatment group only.

### Chlorophyll fluorescence imaging of the seedling leaf

A PAM fluorometer (the MAXI version of IMAGING-PAM; Heinz Walz GmbH, Effeltrich, Germany) was used to capture the images reflecting several chlorophyll fluorescence parameters from a leaf. The imaging was conducted in the dark room (temperature was set at 25 °C). The rice seedlings were dark-adapted at least 30 min before taking measurements on the second leaf of the seedling. To minimize the effect of circadian rhythms on the photosynthesis efficiency, all measurements were taken at the same time during the day. Minimum fluorescence (F_0_) was measured under weak modulating radiation (0.5 μmol m^− 2^ s^− 1^), and maximum fluorescence (F_m_) was recorded by applying a saturating pulse of radiation (2700 μmol m^− 2^ s^− 1^). To measure the light responses, the actinic light (350 μmol m^− 2^ s^− 1^) was switched on to drive photosynthesis for 5 min. Steady-state fluorescence under light illumination (F_t_) was continuously monitored under weak modulating radiation (0.5 μmol m^− 2^ s^− 1^), the maximum fluorescence in the light (F_m_′) was assessed by applying a saturating pulse of radiation (2700 μmol m^− 2^ s^− 1^), and the minimum fluorescence was calculated as F_0_’ = F_0_ / (F_v_/F_m_ + F_0_/F_m_′). These basic measurements were used to derive the relevant fluorescence parameters: F_v_/F_m_, ΦPSII, qL, and NPQ [57].

The actual photosynthetic efficiency [Y(II)] was calculated as described by Genty et al. [58]. The quantum yield of regulated energy dissipation in PSII [Y(NPQ)] and the quantum yield of nonregulated energy dissipation in PSII [Y(NO)] were calculated according to Kramer et al.’s [24] method.

The definition and calculation of six major chlorophyll fluorescence parameters are described below:

1. F_0_ (minimum fluorescence)
2. F_m_ (maximal fluorescence)
3. F_v_/F_m_ (maximal PSII quantum yield), F_v_/F_m_ = (F_m_-F_0_)/F_m_
4. ΦPSII (effective PSII quantum yield), ΦPSII = (F_m_′-F_t_)/F_m_′
5. qL (coefficient of photochemical quenching), qL = (F_m_′-F_t_)/(F_m_′-F_0_’) * (F_0_’/F_t_)
6. NPQ (nonphotochemical quenching), NPQ = (F_m_-F_m_′)/F_m_′
7. Y(II) = (Fm′-Fs)/Fm′
8. Y(NO) = 1/(NPQ + 1 + qL(Fm/Fo-1))
9. Y(NPQ) = 1-Y(II)-1/(NPQ + 1 + qL(Fm/Fo-1))
10. Y(II) + Y(NO) + Y(NPQ) = 1

### Establish a visual standard system to evaluate the salt stress level

To quantify and compare the symptoms of salt toxicity among rice varieties, we modified the International Rice Research Institute (IRRI) standard evaluation system (SES) [59] according to our observations and assigned an injury score on a scale of 1 to 9, where a low score (near 1) represents tolerant individuals and a higher score represents sensitive individuals (Additional file 1: Table S1).

### Genome-wide association analysis

Four statistical models were applied to identify the association between SNPs and trait variations: naïve, P, K, and P + K models. The naïve model was represented by *Y* = *Xβ* + *ε*, where *Y* was phenotype data, *X* was genotype data, β was the SNP effect and ε was random effects. The P model was represented by *Y* = *Xβ* + *Pγ* + *ε*, where *P* was the population structure and γ was the effect of the population structure. The K model was represented by *Y* = *Xβ* + *Zu* + *ε*, where u was the random effect of kinship and Z was a coincidence matrix. The P + K model was represented by *Y* = *Xβ* + *Pγ* + *Zu* + *ε*. The population structure was analyzed by principal component analysis [60]. Association analyses were conducted in TASSEL 4.0 [61] and R/GAPIT [62].

The LD between the most significant SNP and its neighboring SNPs was estimated using Tagger in Haploview 4.2 [63]. The candidate region was determined based on the LD (r^2^ > 0.7) between the most significant SNP and its neighboring SNPs, and the candidate genes within the region were identified using Gramene BioMart (http://ensembl.gramene.org/biomart). The SNP position was based on IRGSP 1.0 annotation.

### Statistical analysis

Pearson correlation was used to estimate the level of correlation between traits. The normality of each trait was tested using the Shapiro-Wilk method. The genetic distance between the diverse accessions was calculated using the “euclidean” method, and agglomerative hierarchical clustering using the “complete” method was applied to evaluate the relationship between traits and rice accession genetic backgrounds and was plotted as a heatmap. All analyses were conducted using publicly available R packages.

## Additional files

Additional file 1: **Table S1.** Phenotypic evaluation to score the visual symptoms of salt toxicity at the seedling stage (DOCX 14 kb)

Additional file 2: **Table S2.** The quantum yield calculated from the ratio of treatment to control (T/C) values of each parameter in eight varieties (XLSX 14 kb)

Additional file 3: **Table S3.** Chlorophyll fluorescence and injury score of 232 accessions and their subpopulation assignment (XLSX 33 kb)

Additional file 4: **Table S4.** Correlation between chlorophyll florescence parameters and injury score (IS) in the global panel (232 varieties) (DOCX 16 kb)

Additional file 5: **Table S5.** The genes inside the LD region of the most significant SNP detected in the F_0_ GWAS (XLSX 75 kb)

Additional file 6: **Table S6.** The genes inside the LD region of the most significant SNP detected in the qL GWAS (XLSX 112 kb)

Additional file 7: **Figure S1.** Daily changes in chlorophyll fluorescence parameters (ΦPSII, qL, NPQ) in eight rice varieties under control (red line) and salt (blue line) conditions. Each data point represents the average value of 3 replicates. (PPTX 10924 kb)

Additional file 8: **Figure S2.** Box plot of six chlorophyll fluorescence measurements and injury score in six subpopulations and two subspecies. Student’s t-test was used to determine whether any two subpopulations or two subspecies were different. *, **, and **** represent the significance level at *P* < 0.05, 0.01 and 0.0001, respectively. (PDF 979 kb)

Additional file 9: **Figure S3.** Genetic relationship and phenotypic distributions of varieties in five subpopulations. Each column represents a single variety, and the sample ID is labeled at the bottom of the heatmap. Each row represents the chlorophyll fluorescence parameter and injury score of each variety. The colors close to red indicate that the value of each chlorophyll fluorescence parameter is higher than its average value over all accessions; for the injury score, the red represents the lower injury scores, which suggest that the variety is more tolerant to salt toxicity. The color key of the Z-score was calculated from the distance between the raw score and the population mean in units of the standard deviation. (PDF 1686 kb)

Additional file 10: **Figure S4.** Distribution of six chlorophyll fluorescence parameters in *Japonica* varieties. The normality of each parameter was examined using the Shapiro-Wilks test. The *P*-value of each test is provided. (PPTX 7609 kb)

Additional file 11: **Figure S5.** Distribution of six chlorophyll fluorescence parameters in *Indica* varieties. The normality of each parameter was examined using the Shapiro-Wilks test. The *P*-value of each test is provided. (PPTX 7606 kb)

Additional file 12: **Figure S6.** Genome-wide association analysis of six chlorophyll fluorescence parameters in 232 diverse varieties or in two subspecies (*Japonica* and *Indica*). Manhattan plot showing the significance of each SNP tested by the appropriate statistical models described in “Methods”. (PDF 4610 kb)

Additional file 13: ** Figure S7.** Genome-wide association analysis of qL in 232 diverse varieties and LD pattern of the most significant SNP. (a) Manhattan plot showing the significance of each SNP tested by a mixed linear model. (b) The most significant SNP is at 26,473,167 bp. The dot color of each SNP represents its LD with the most significant SNP, and the decay of LD is bordered by two vertical dashed lines. The location of the *OsCBL4* gene is marked with a blue bar. (PPTX 14234 kb)

## Data Availability

All data generated or analyzed during this study are included in this published article and its supplementary information files.

## Abbreviations

ETR: Electron transfer rate
GWAS: Genome-wide association study
IRRI: International Rice Research Institute
LD: Linkage disequilibrium
LHC: Light-harvesting complex
NPQ: Nonphotochemical quenching
PAM: Pulse-amplitude modulation
PS: Photosystem
*Q-Q*: Quantile-quantile
QTL: Quantitative trait loci
RGR: Relative growth rate
SES: Standard evaluation system
*TEJ*: *Temperate japonica*
TR: Transpiration rate
TRJ: *Tropical japonica*
TUE: Transpiration use efficiency
