# Supplementary material for: Chlorophyll fluorescence analysis in diverse rice varieties reveals the positive correlation between the seedlings salt tolerance and photosynthetic efficiency
Source: BMC Plant Biol. 2019 Sep 13;19:403. doi: 10.1186/s12870-019-1983-8 (PMC6743182; doi:10.1186/s12870-019-1983-8)
Supplement: Supplementary file 1 — Table S1. Phenotypic evaluation to score the visual symptoms of salt toxicity at the seedling stage (DOCX 14 kb) [file 12870_2019_1983_MOESM1_ESM.docx]

| Table S1. Phenotypic evaluation to score the visual symptoms of salt toxicity at seedling stage | |
| --- | --- |
| Injury Score | Observed seedling phenotype |
| 1 | Normal and healthy growth |
| 3 | Half of second leaf is whitish, or tip of 3rd leaf turn whitish or rolled |
| 5 | All 2nd leaf whitish or rolled, or half of 3rd leaf show whitish or rolled |
| 7 | At least two-third of 3rd leaf turn white or rolled. |
| 9 | Entire plant dead or dying. |
