# Supplementary material for: Chlorophyll fluorescence analysis in diverse rice varieties reveals the positive correlation between the seedlings salt tolerance and photosynthetic efficiency
Source: BMC Plant Biol. 2019 Sep 13;19:403. doi: 10.1186/s12870-019-1983-8 (PMC6743182; doi:10.1186/s12870-019-1983-8)
Supplement: Supplementary file 4 — Table S4. Correlation between chlorophyll florescence parameters and injury score (IS) in the global panel (232 varieties) (DOCX 16 kb) [file 12870_2019_1983_MOESM4_ESM.docx]

| Table S4. Correlation between chlorophyll florescence parameters and injury score (IS) in global diversity panel (232 varieties). | | | | | | | |
| --- | --- | --- | --- | --- | --- | --- | --- |
| The lower triangle shows the Pearson's correlation coefficient (r). The upper triangle shows the *P* value of the correlation. *P*-value was determined by two tailed Student's *t*-test. | | | | | | | |
|  | F0 | Fm | Fv/Fm | ΦPSII | qL | NPQ | IS |
| F0 | 1 | <0.001 | 0.9134 | 0.3013 | 0.008331 | <0.001 | 0.2105 |
| Fm | 0.36207 | 1 | <0.001 | <0.001 | <0.001 | <0.001 | <0.001 |
| Fv/Fm | 0.007178 | 0.830823 | 1 | <0.001 | <0.001 | <0.001 | <0.001 |
| ΦPSII | -0.06816 | 0.728402 | 0.718819 | 1 | <0.001 | <0.001 | <0.001 |
| qL | 0.172845 | 0.549629 | 0.488726 | 0.853551 | 1 | <0.001 | <0.001 |
| NPQ | 0.275949 | 0.804982 | 0.729973 | 0.611289 | 0.61837 | 1 | <0.001 |
| IS | 0.057084 | -0.48065 | -0.45771 | -0.52299 | -0.45094 | -0.39553 | 1 |
