# Supplementary material for: Chlorophyll fluorescence analysis in diverse rice varieties reveals the positive correlation between the seedlings salt tolerance and photosynthetic efficiency
Source: BMC Plant Biol. 2019 Sep 13;19:403. doi: 10.1186/s12870-019-1983-8 (PMC6743182; doi:10.1186/s12870-019-1983-8)
Supplement: Supplementary file 7 — Figure S1. Daily changes in chlorophyll fluorescence parameters (ΦPSII, qL, NPQ) in eight rice varieties under control (red line) and salt (blue line) conditions. Each data point represents the average value of 3 replicates. (PPTX 10924 kb) [file 12870_2019_1983_MOESM7_ESM.pptx]

## Slide 1
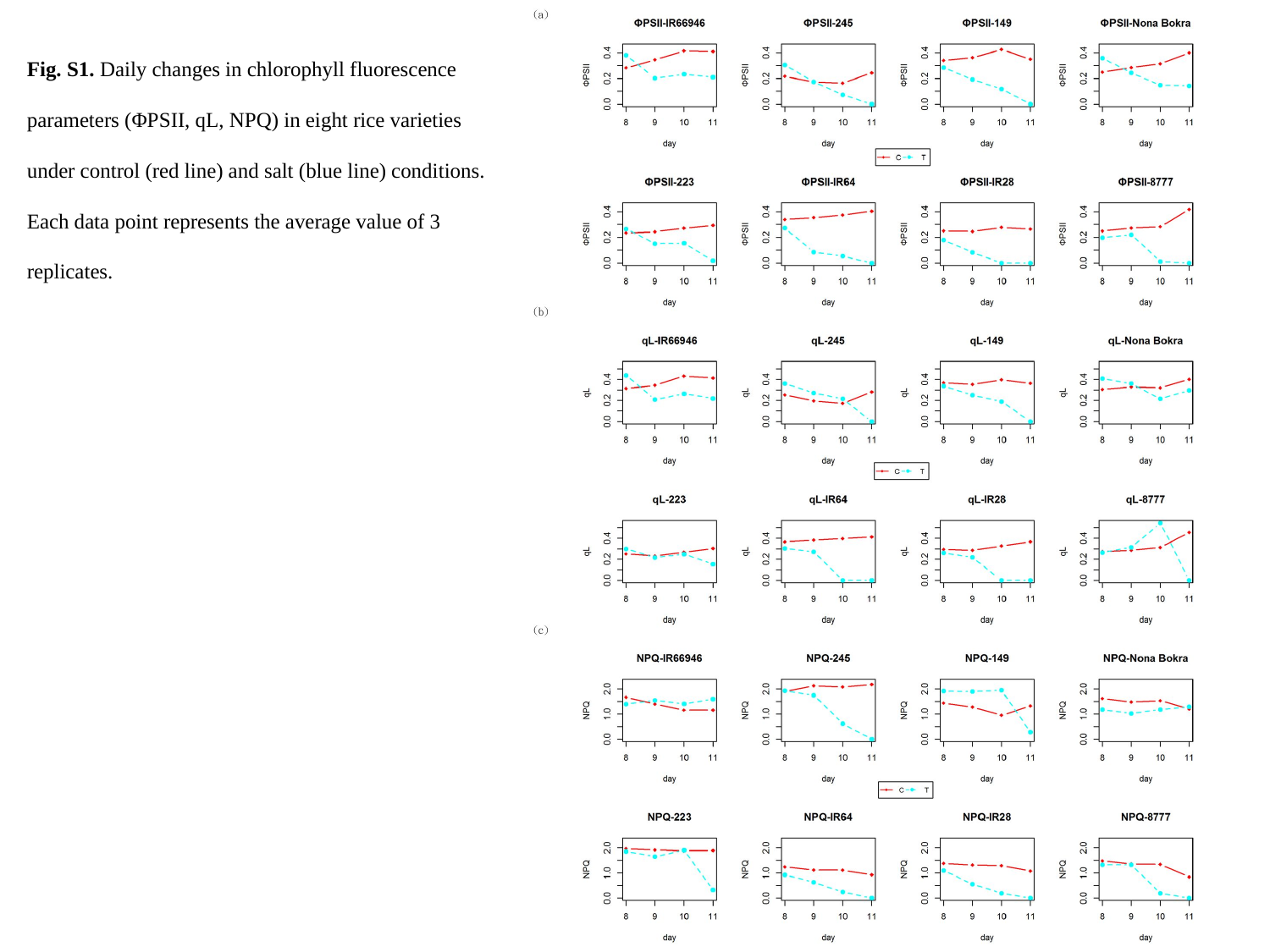

Fig. S1. Daily changes in chlorophyll fluorescence parameters (ΦPSII, qL, NPQ) in eight rice varieties under control (red line) and salt (blue line) conditions. Each data point represents the average value of 3 replicates.
