# Supplementary figures and images for: Chlorophyll fluorescence analysis in diverse rice varieties reveals the positive correlation between the seedlings salt tolerance and photosynthetic efficiency
Source: BMC Plant Biol. 2019 Sep 13;19:403. doi: 10.1186/s12870-019-1983-8 (PMC6743182; doi:10.1186/s12870-019-1983-8)

(a)

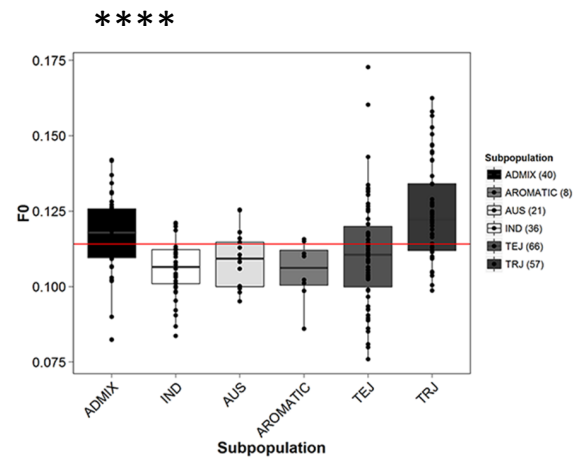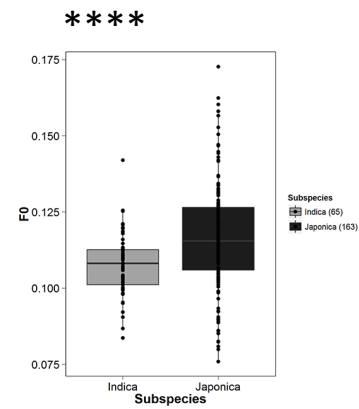

(b)

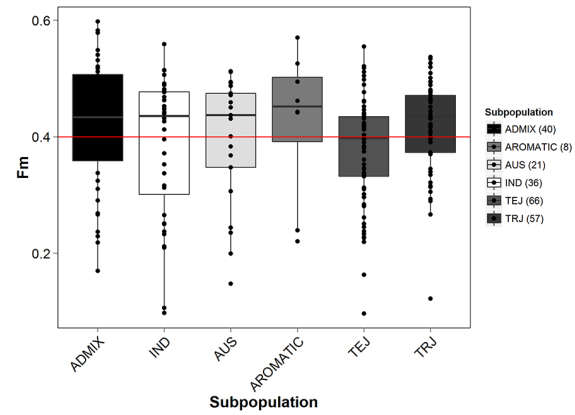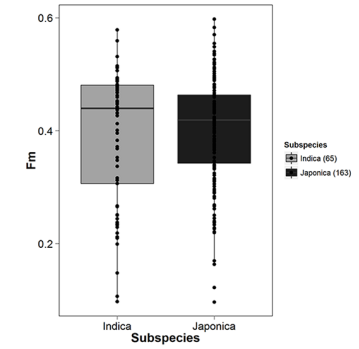

(c)

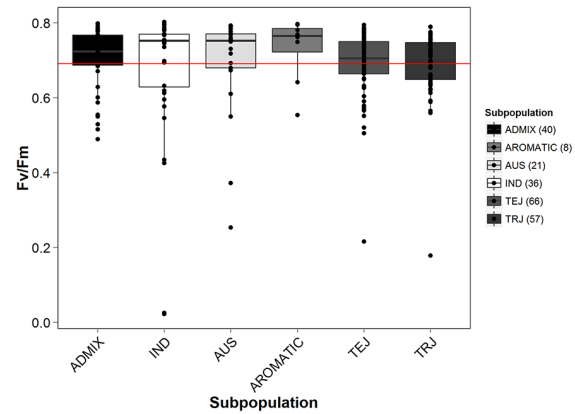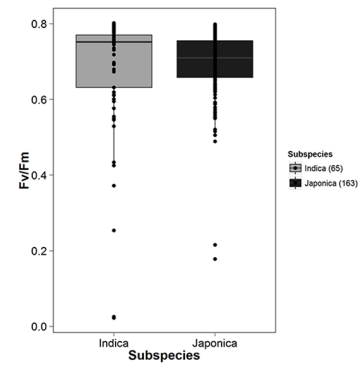

(d)

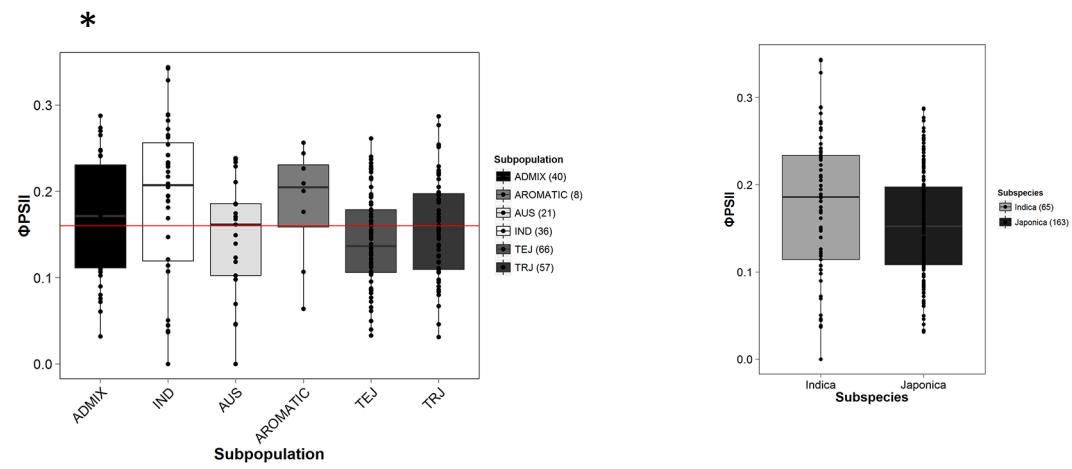

(e)

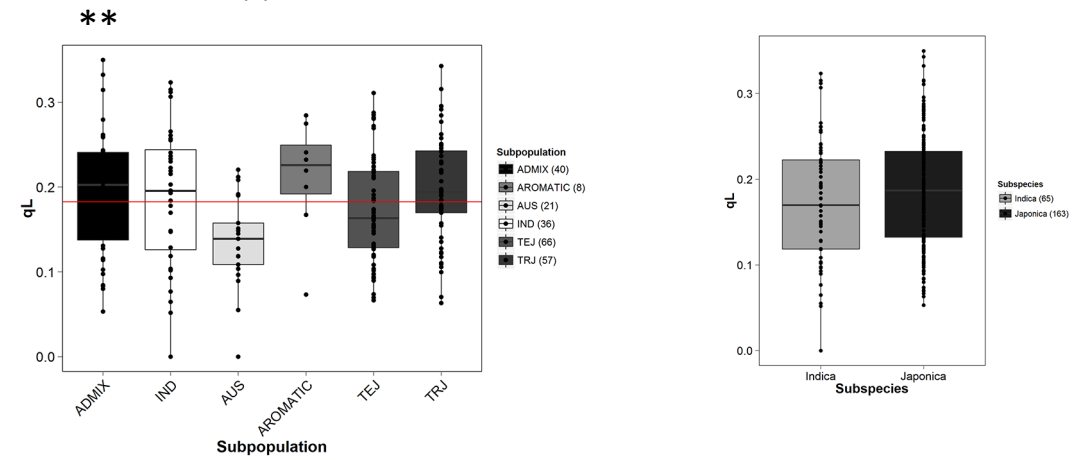

(f)

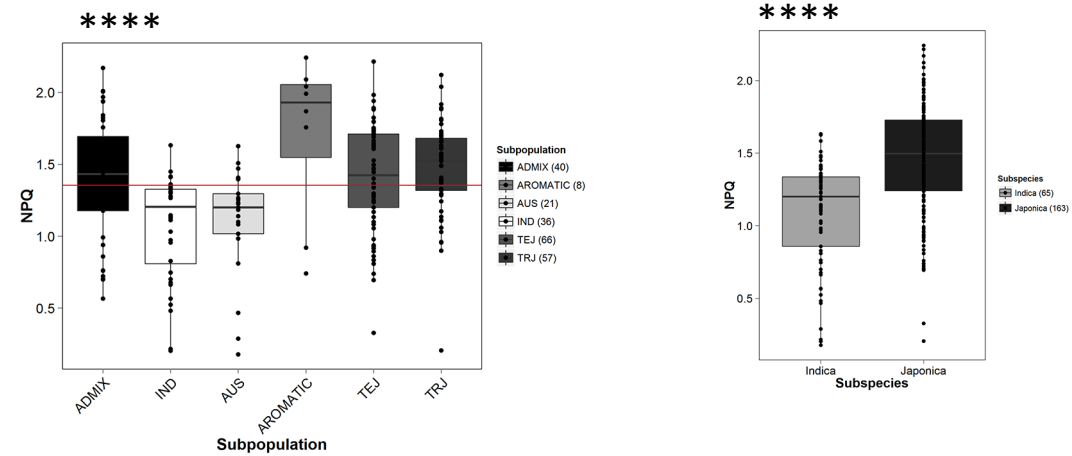

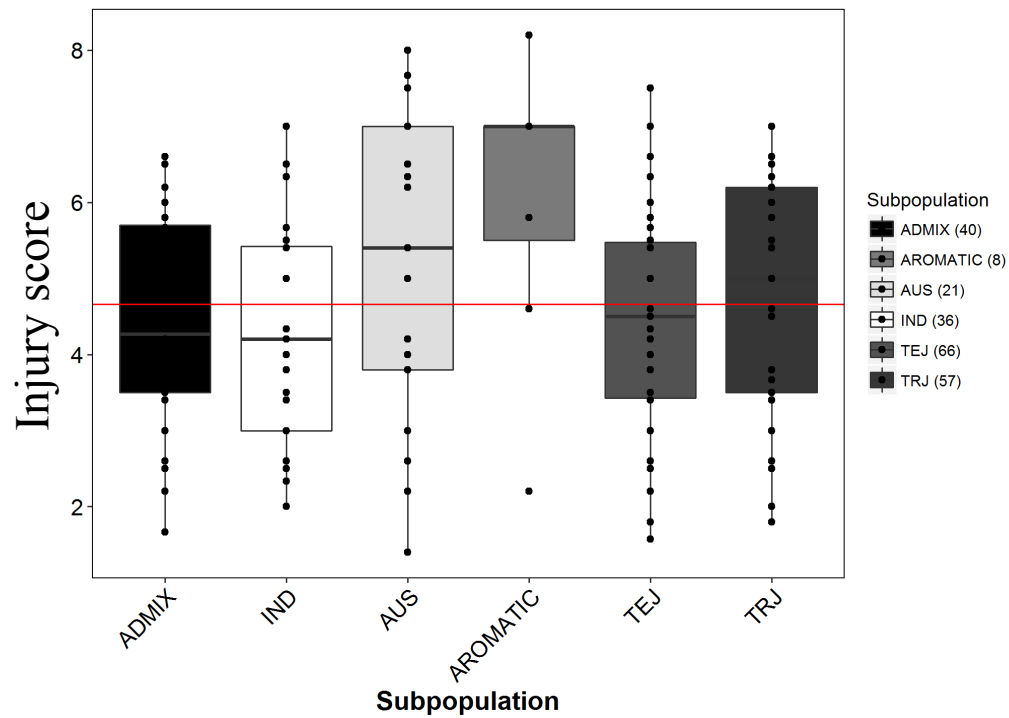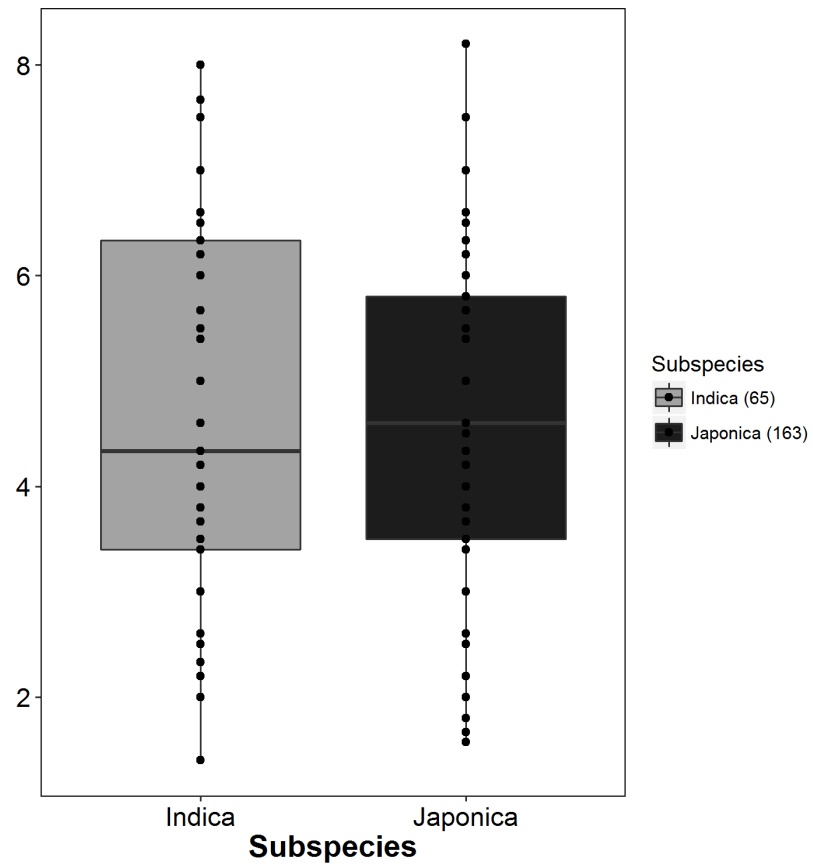

Supplement: Supplementary file 8 — Figure S2. Box plot of six chlorophyll fluorescence measurements and injury score in six subpopulations and two subspecies. Student’s t-test was used to determine whether any two subpopulations or two subspecies were different. *, **, and **** represent the significance level at P < 0.05, 0.01 and 0.0001, respectively. (PDF 979 kb) [file 12870_2019_1983_MOESM8_ESM.pdf]

*Tropical japonica*

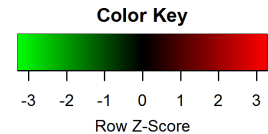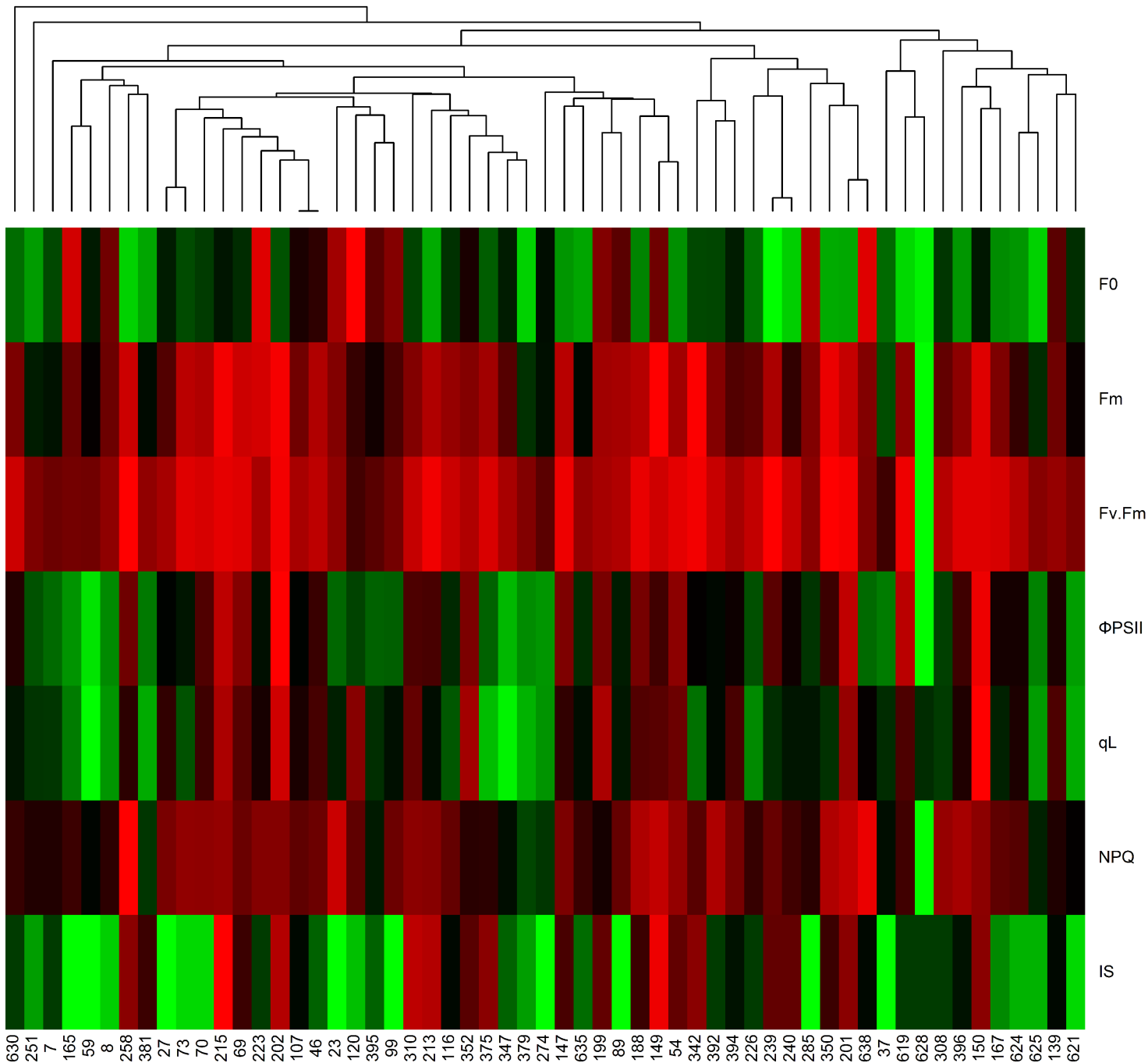

*Aromatic*

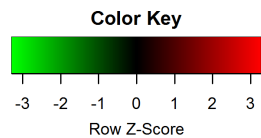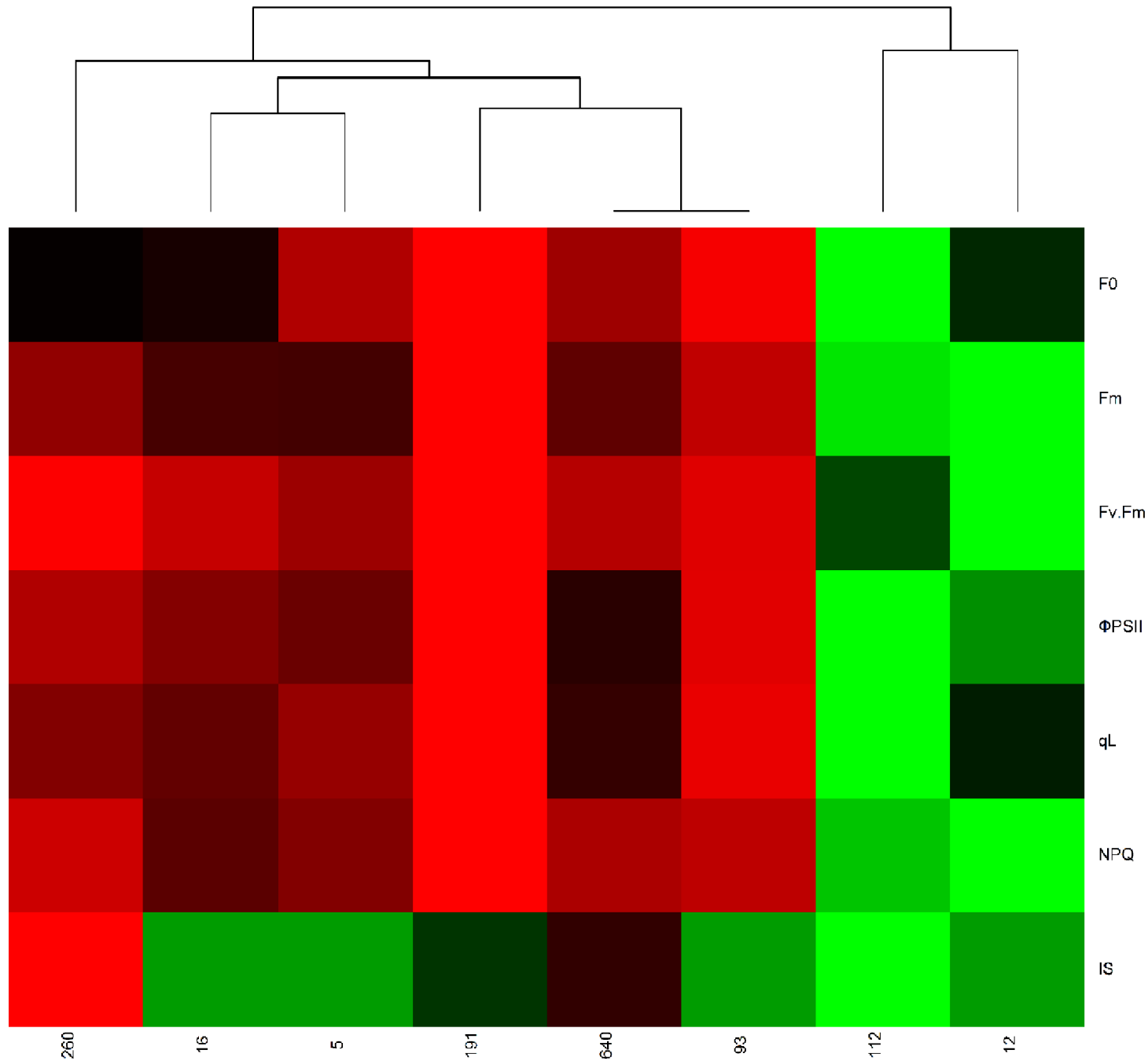

*Indica*

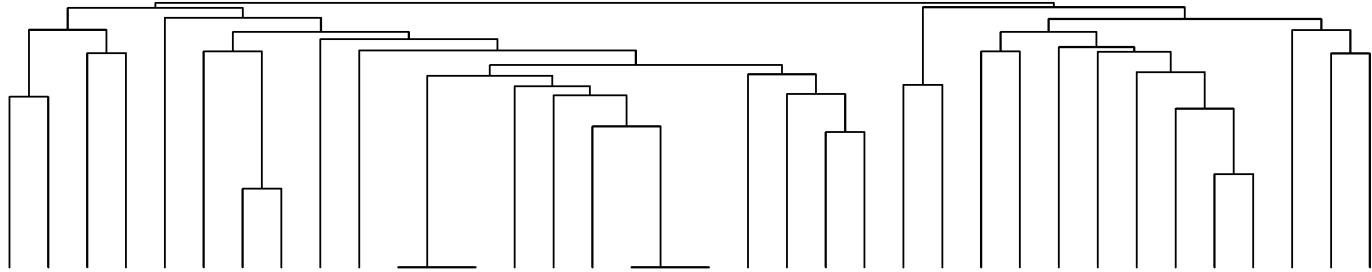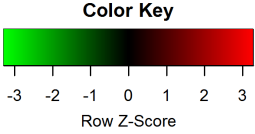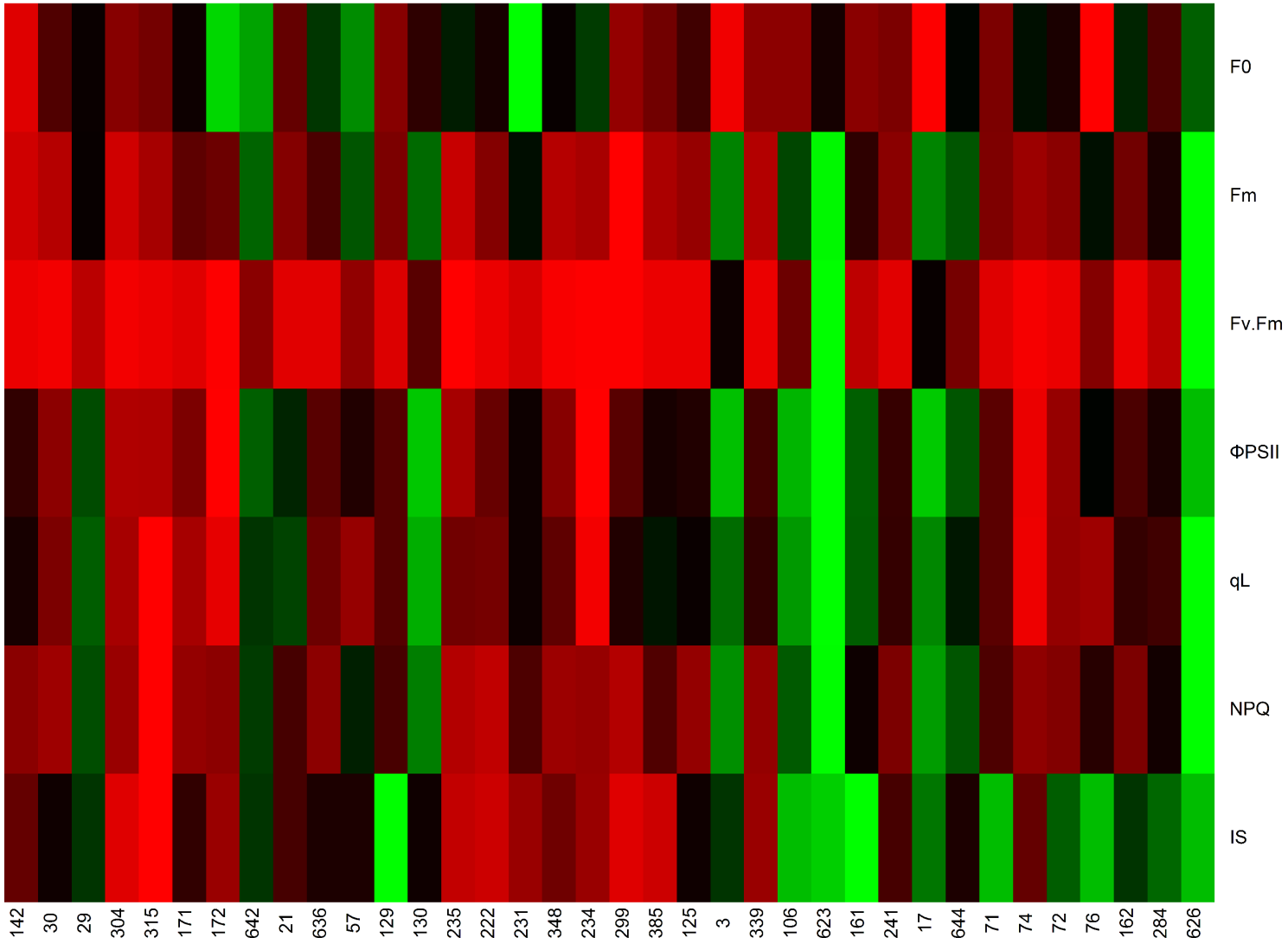

*Aus*

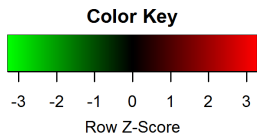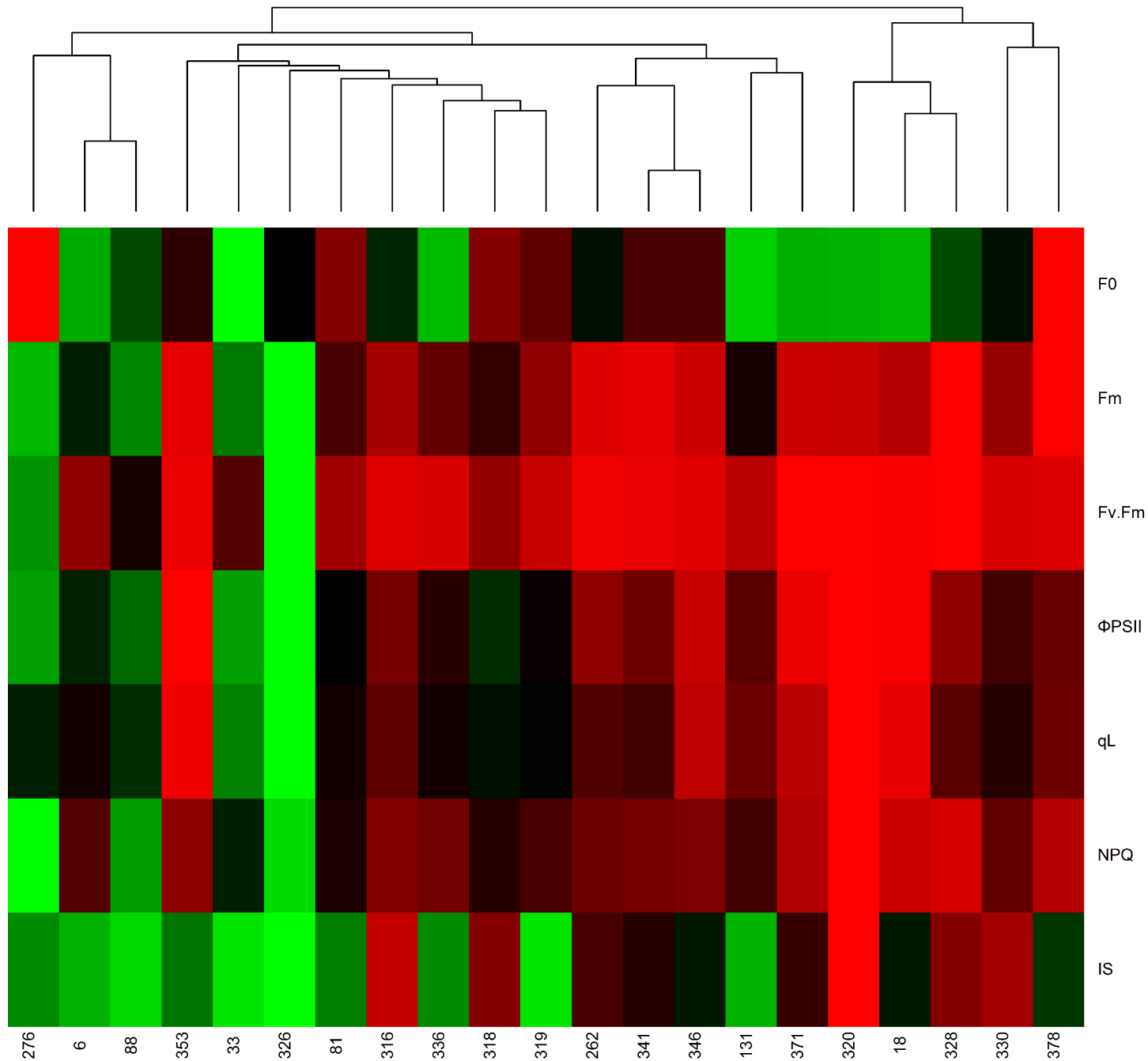

Admix

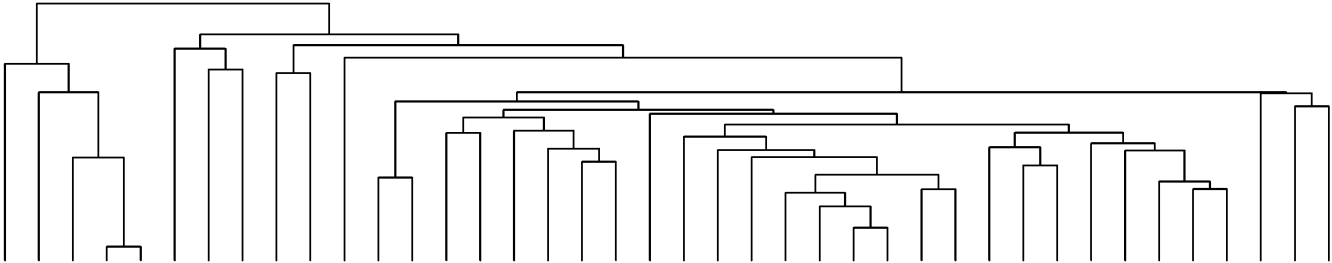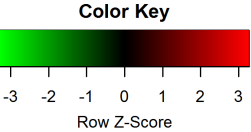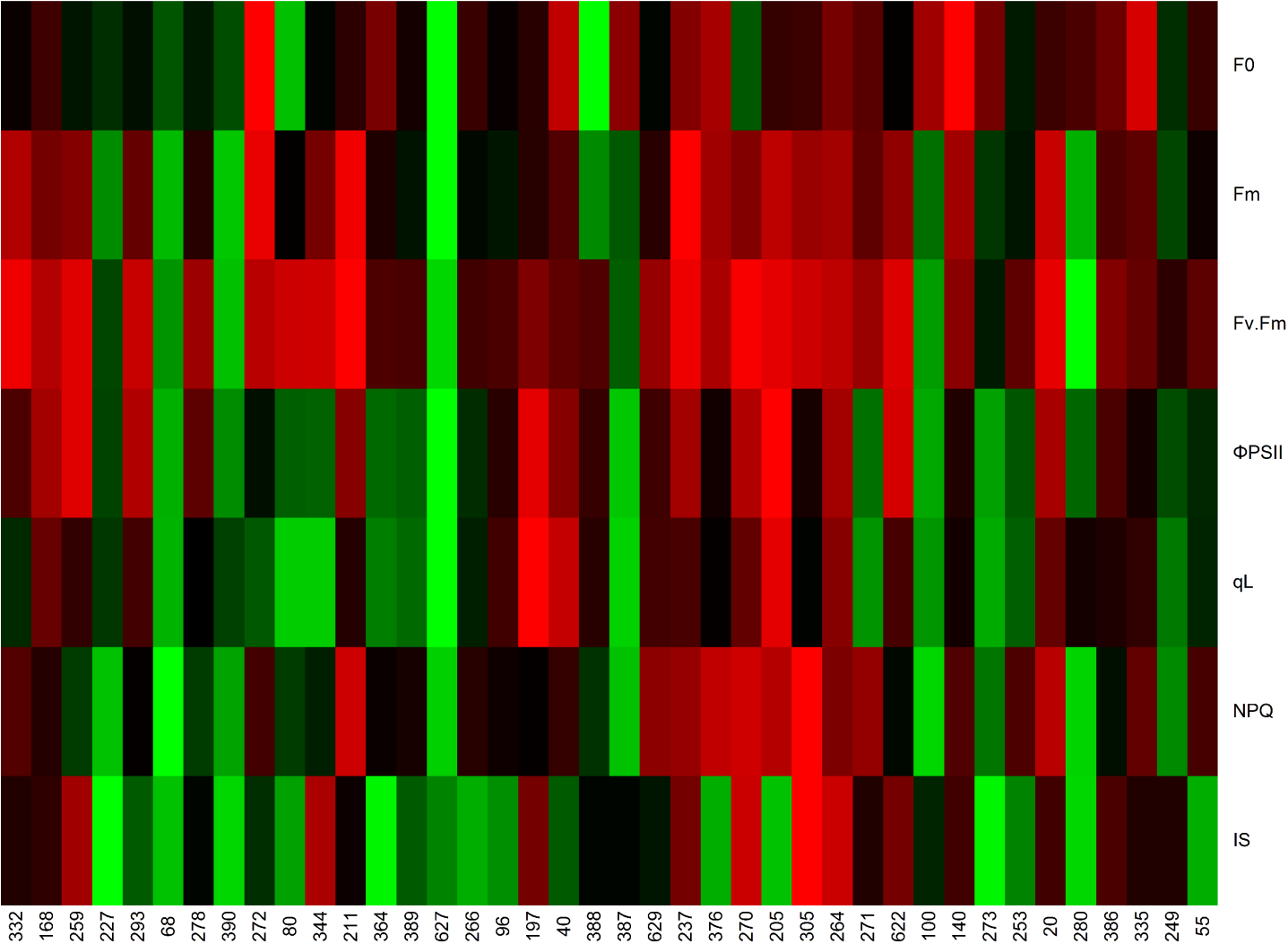

Supplement: Supplementary file 9 — Figure S3. Genetic relationship and phenotypic distributions of varieties in five subpopulations. Each column represents a single variety, and the sample ID is labeled at the bottom of the heatmap. Each row represents the chlorophyll fluorescence parameter and injury score of each variety. The colors close to red indicate that the value of each chlorophyll fluorescence parameter is higher than its average value over all accessions; for the injury score, the red represents the lower injury scores, which suggest that the variety is more tolerant to salt toxicity. The color key of the Z-score was calculated from the distance between the raw score and the population mean in units of the standard deviation. (PDF 1686 kb) [file 12870_2019_1983_MOESM9_ESM.pdf]

## Slide 1
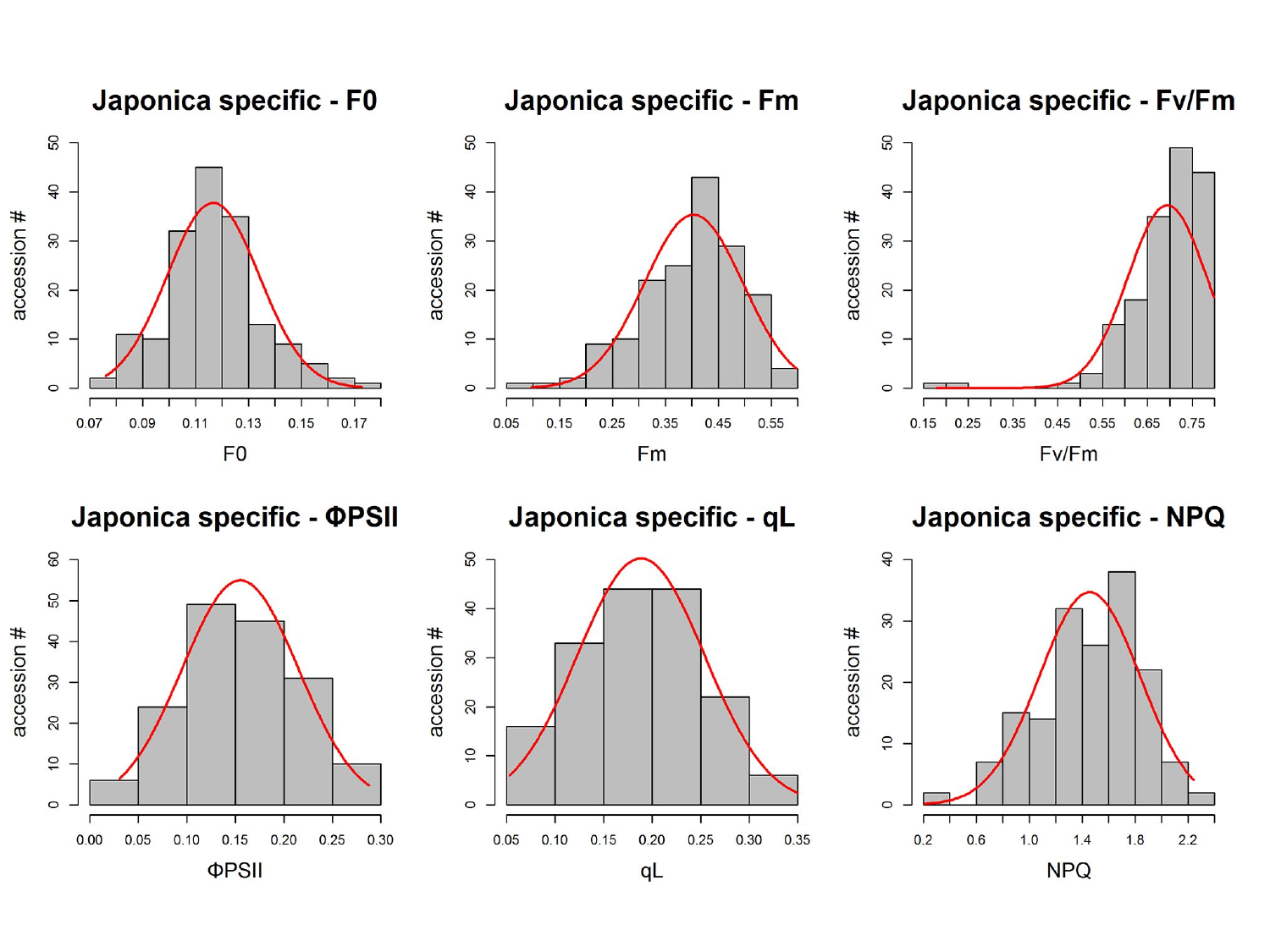

Supplement: Supplementary file 10 — Figure S4. Distribution of six chlorophyll fluorescence parameters in Japonica varieties. The normality of each parameter was examined using the Shapiro-Wilks test. The P-value of each test is provided. (PPTX 7609 kb) [file 12870_2019_1983_MOESM10_ESM.pptx]

## Slide 1
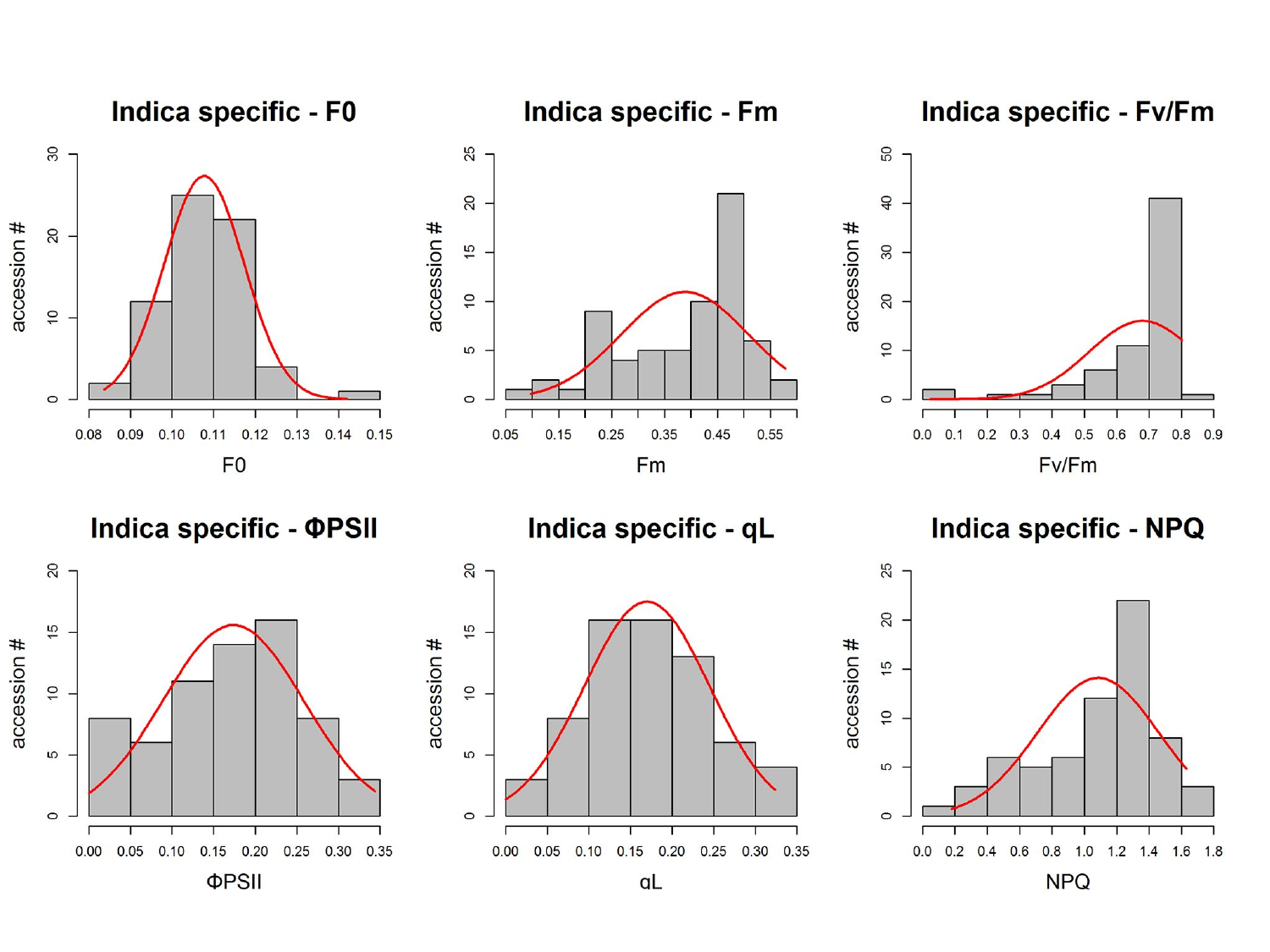

Supplement: Supplementary file 11 — Figure S5. Distribution of six chlorophyll fluorescence parameters in Indica varieties. The normality of each parameter was examined using the Shapiro-Wilks test. The P-value of each test is provided. (PPTX 7606 kb) [file 12870_2019_1983_MOESM11_ESM.pptx]

## Slide 1
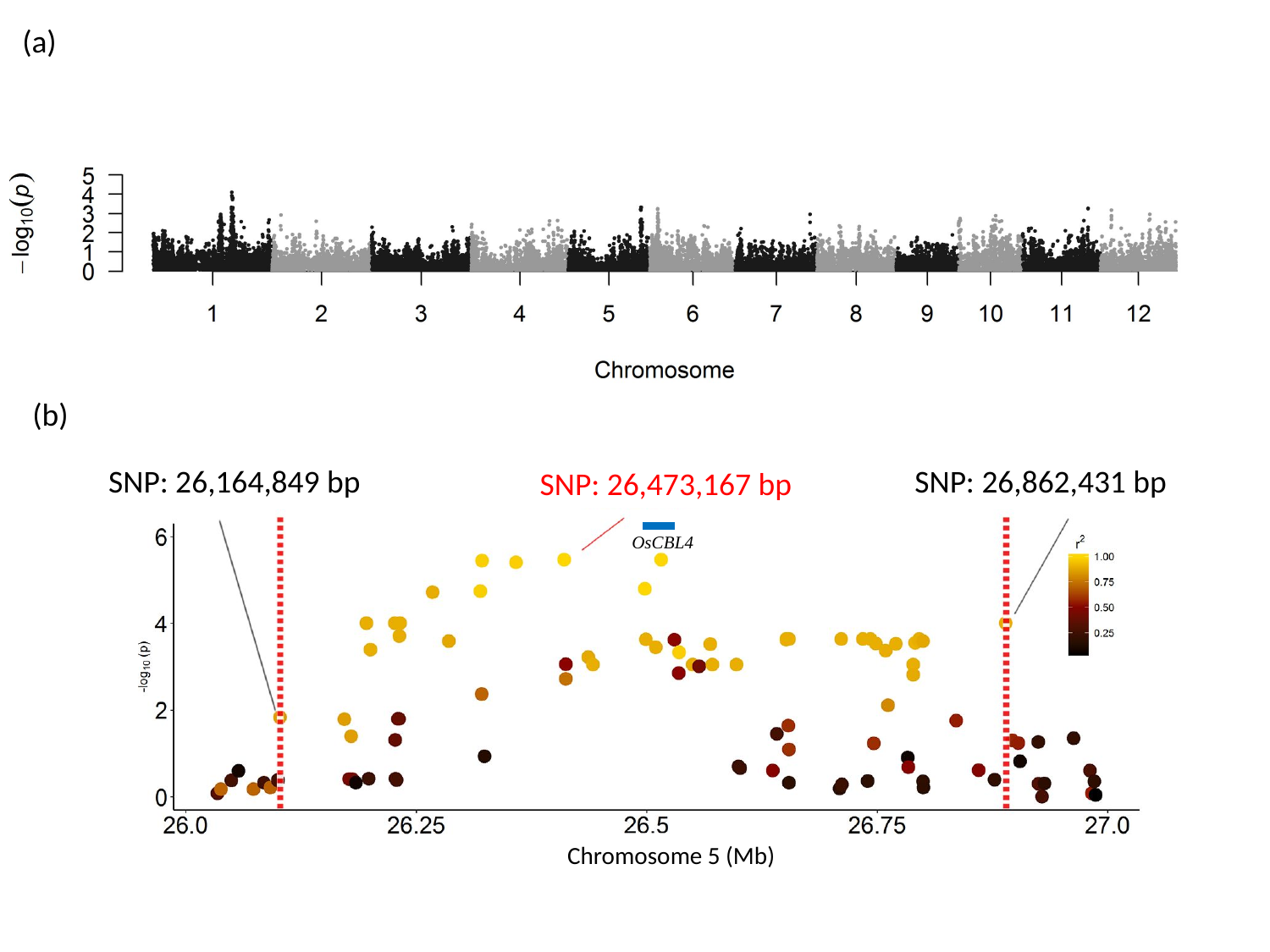

(a)
(b)
SNP: 26,164,849 bp
SNP: 26,862,431 bp
SNP: 26,473,167 bp
OsCBL4
Chromosome 5 (Mb)

Supplement: Supplementary file 13 — Figure S7. Genome-wide association analysis of qL in 232 diverse varieties and LD pattern of the most significant SNP. (a) Manhattan plot showing the significance of each SNP tested by a mixed linear model. (b) The most significant SNP is at 26,473,167 bp. The dot color of each SNP represents its LD with the most significant SNP, and the decay of LD is bordered by two vertical dashed lines. The location of the OsCBL4 gene is marked with a blue bar. (PPTX 14234 kb) [file 12870_2019_1983_MOESM13_ESM.pptx]
