# Supplementary material for: Chlorophyll fluorescence analysis in diverse rice varieties reveals the positive correlation between the seedlings salt tolerance and photosynthetic efficiency
Source: BMC Plant Biol. 2019 Sep 13;19:403. doi: 10.1186/s12870-019-1983-8 (PMC6743182; doi:10.1186/s12870-019-1983-8)

F_0 – whole panel

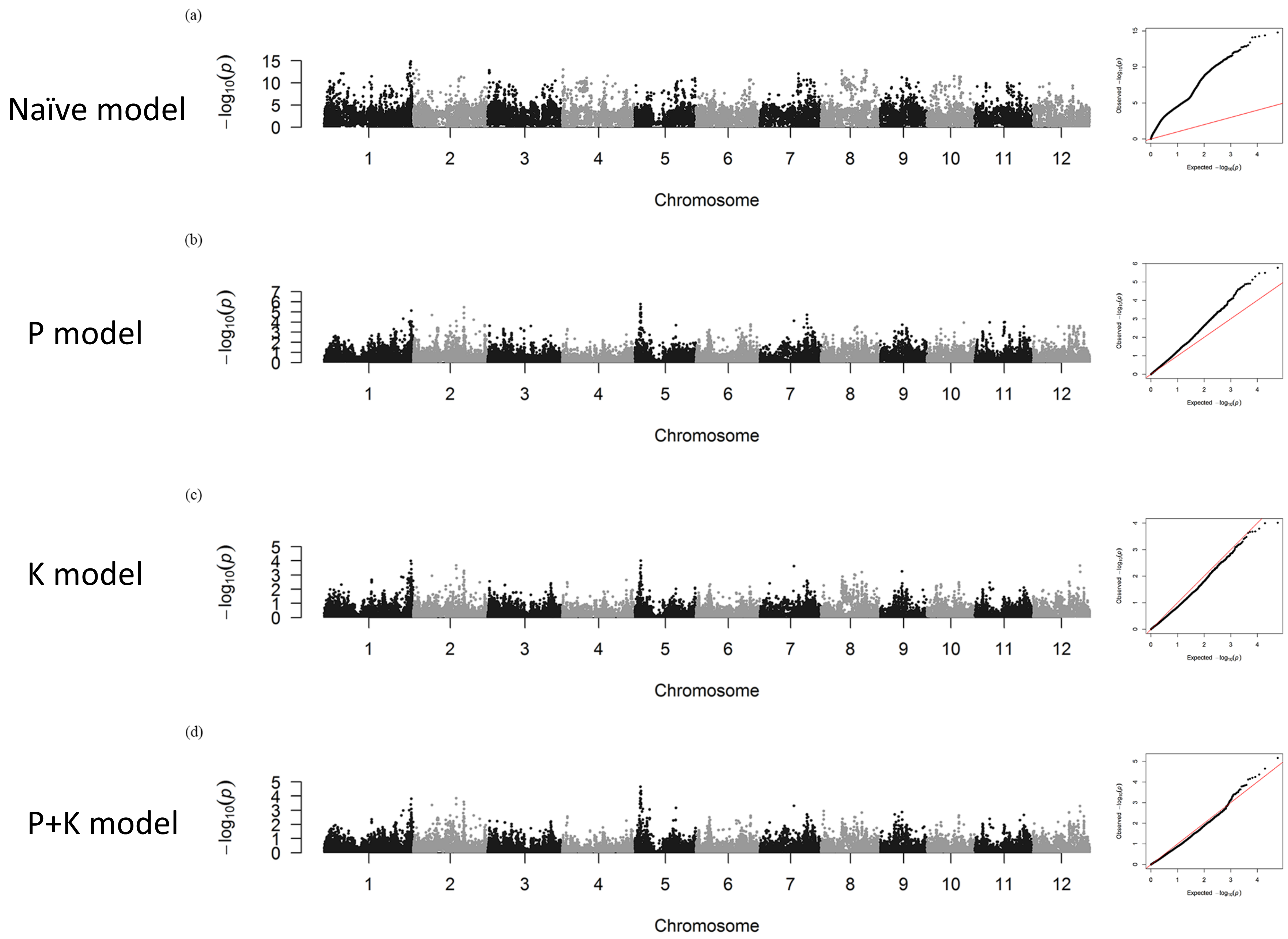

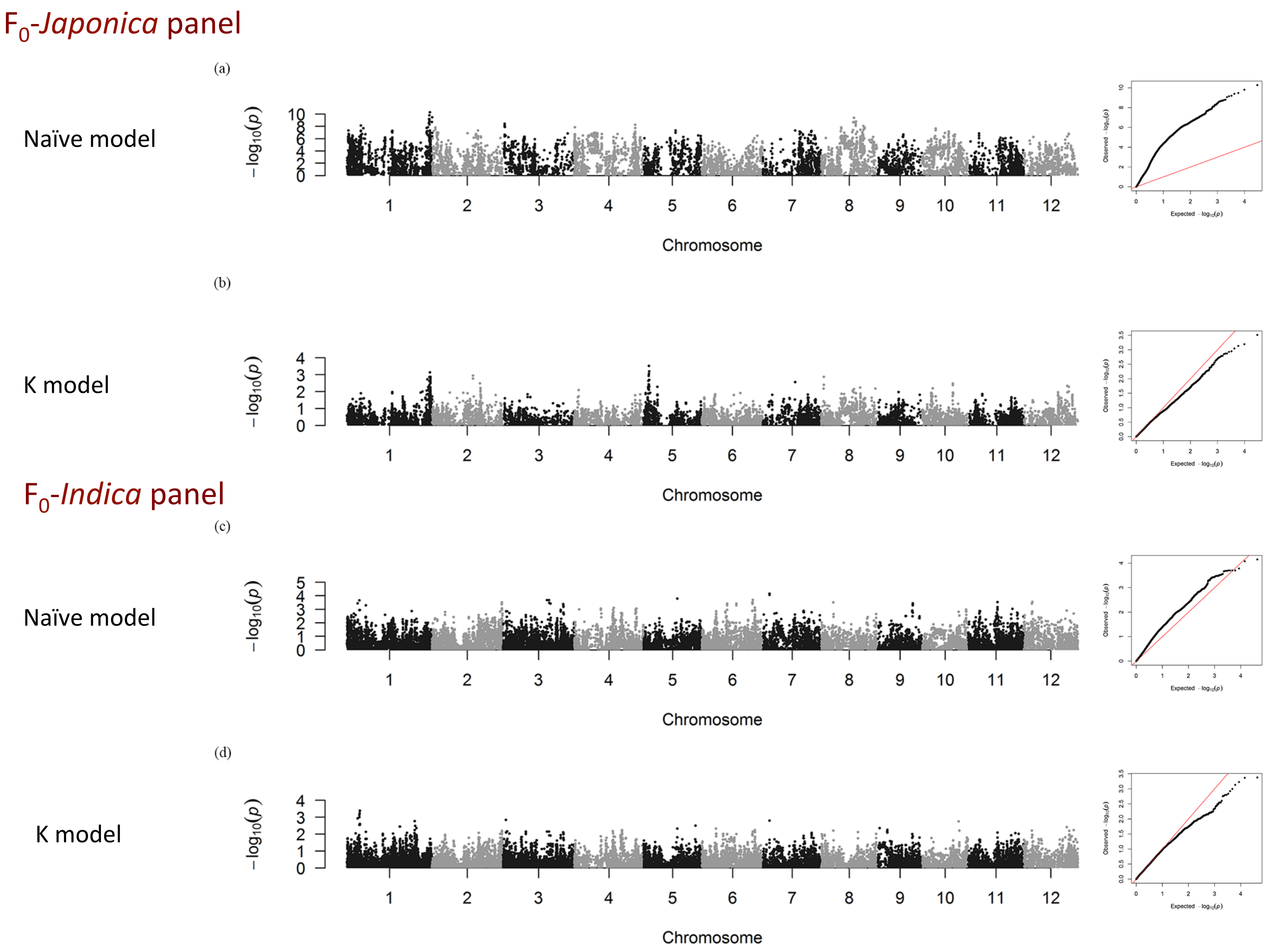

F0-Japonica panel
(a)
Naïve model
−log10(p)
Chromosome
Observed −log10(p)
Expected −log10(p)
(b)
K model
−log10(p)
Chromosome
Observed −log10(p)
Expected −log10(p)
F0-Indica panel
(c)
Naïve model
−log10(p)
Chromosome
Observed −log10(p)
Expected −log10(p)
(d)
K model
−log10(p)
Chromosome
Observed −log10(p)
Expected −log10(p)

F_m – whole panel

Naïve model

P model

K model

P+K model

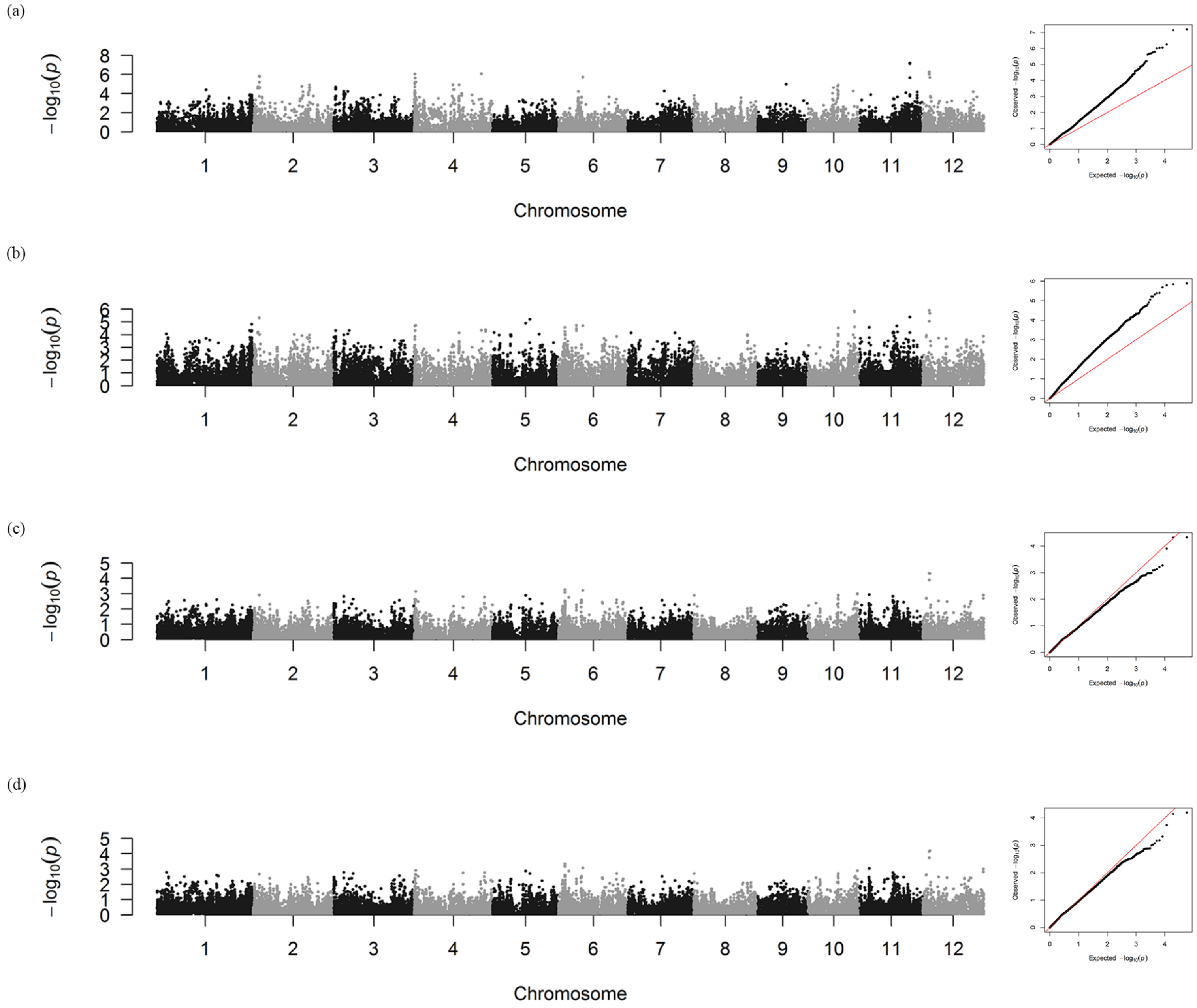

Fm – *Japonica* panel

Naïve model

K model

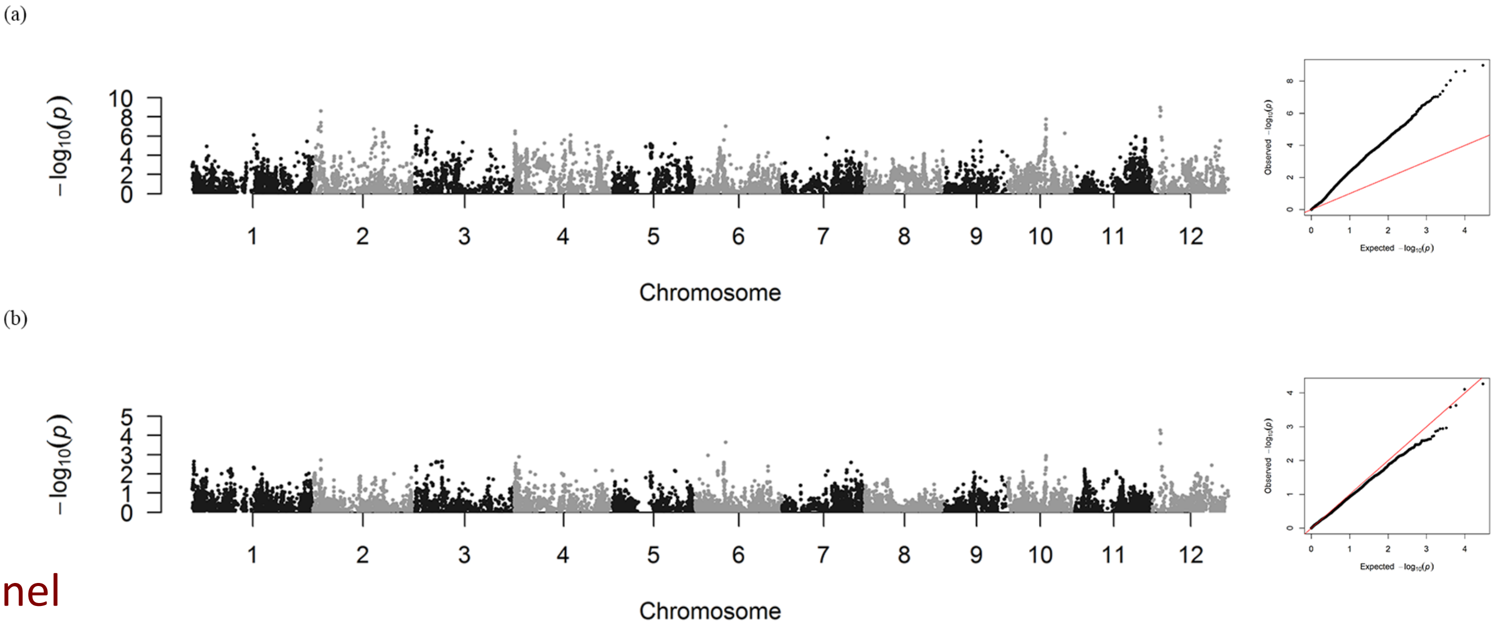

Fm – *Indica* panel

Naïve model

K model

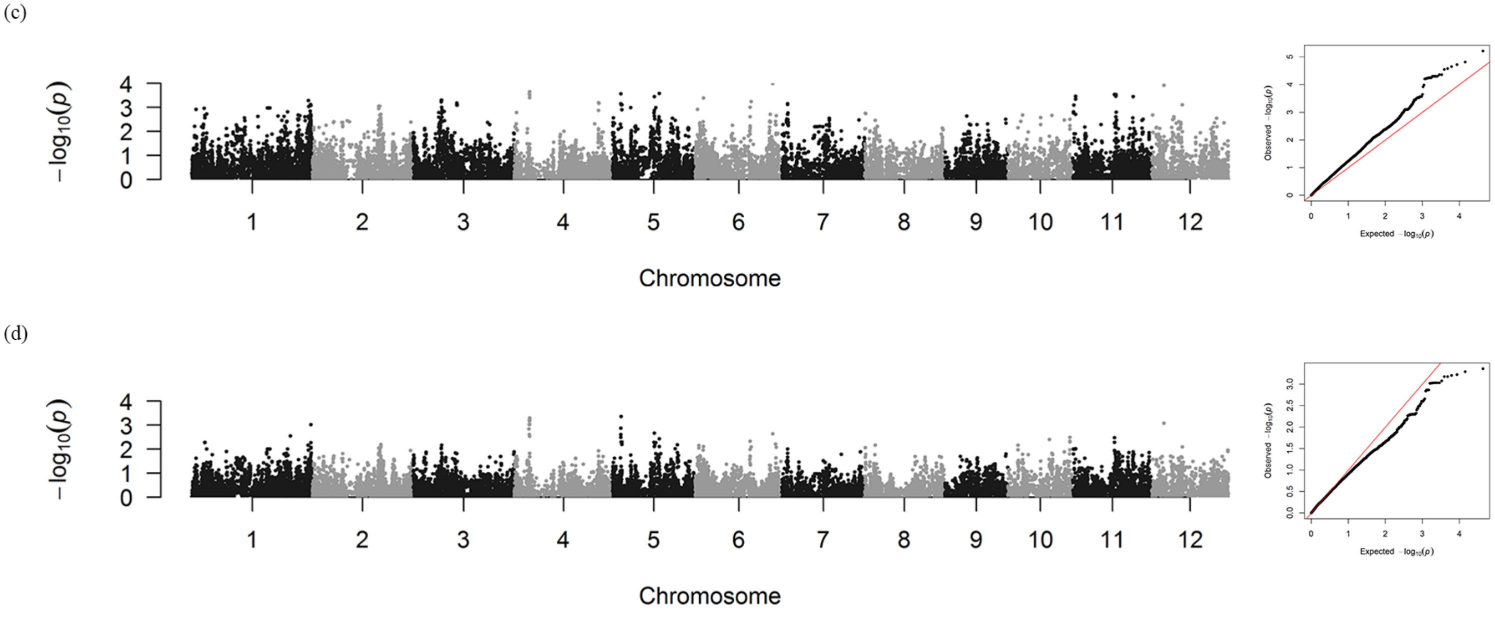

Φ PSII – whole panel

Naïve model

P model

K model

P+K model

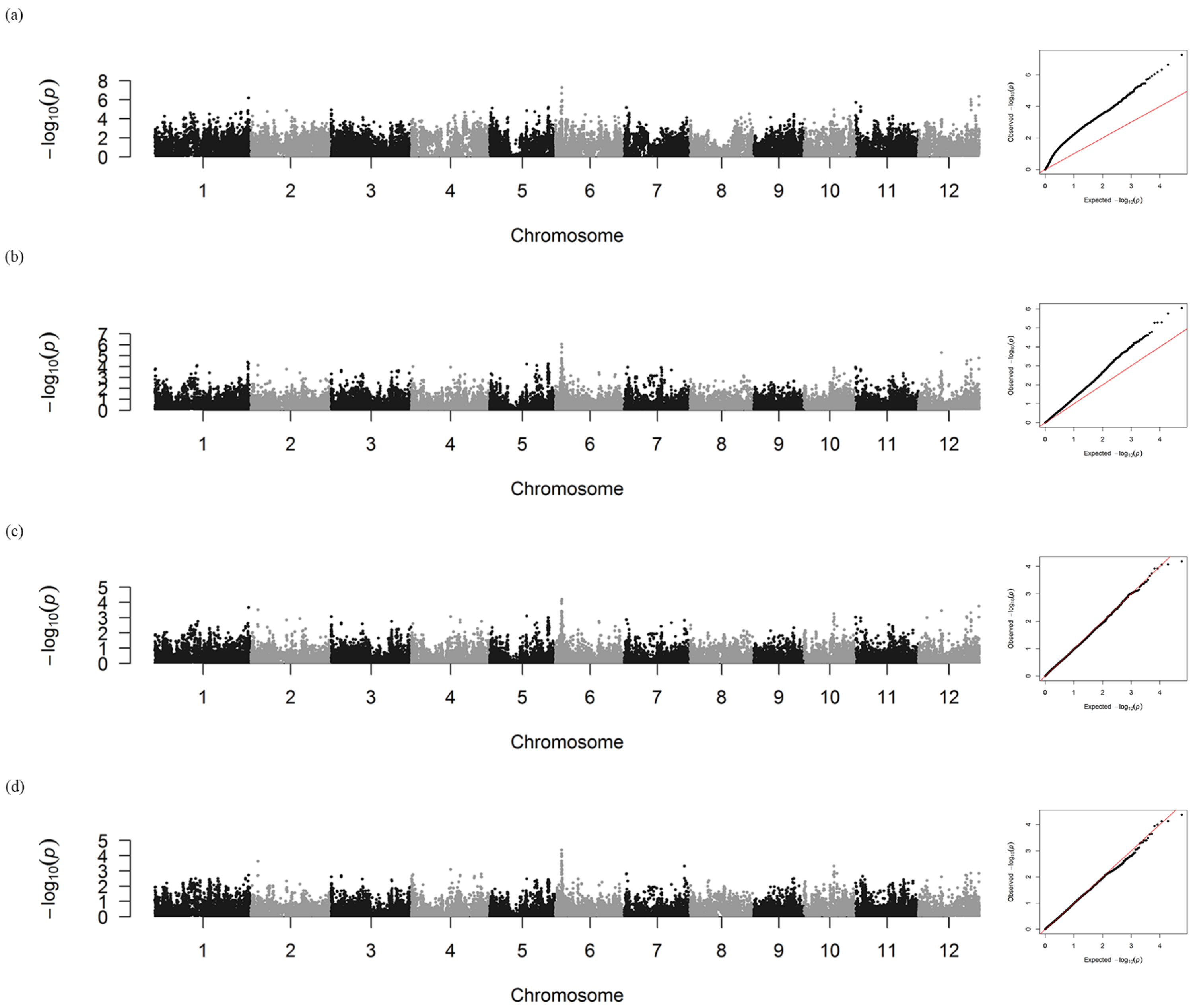

ΦPSII-*Japonica* panel

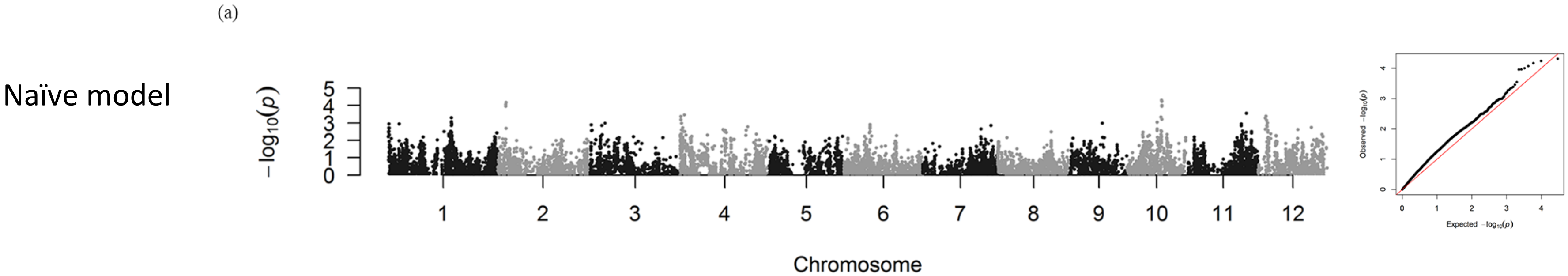

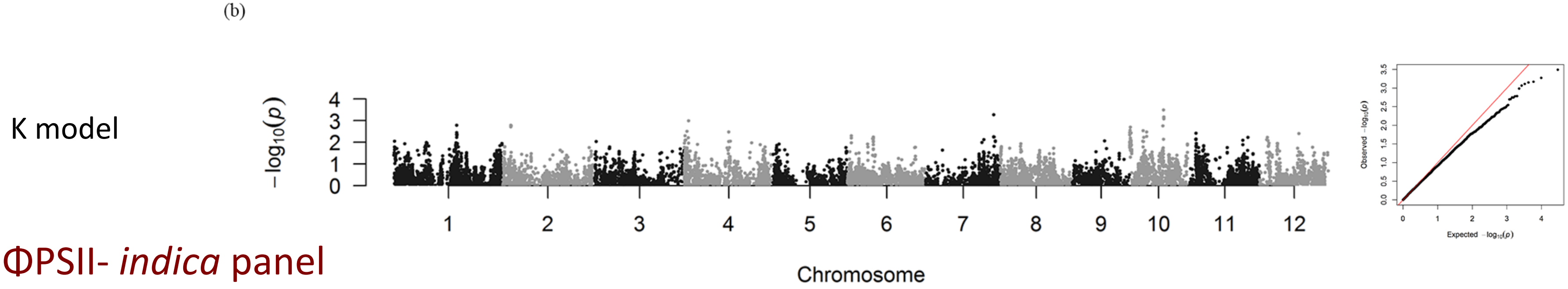

ΦPSII- *indica* panel

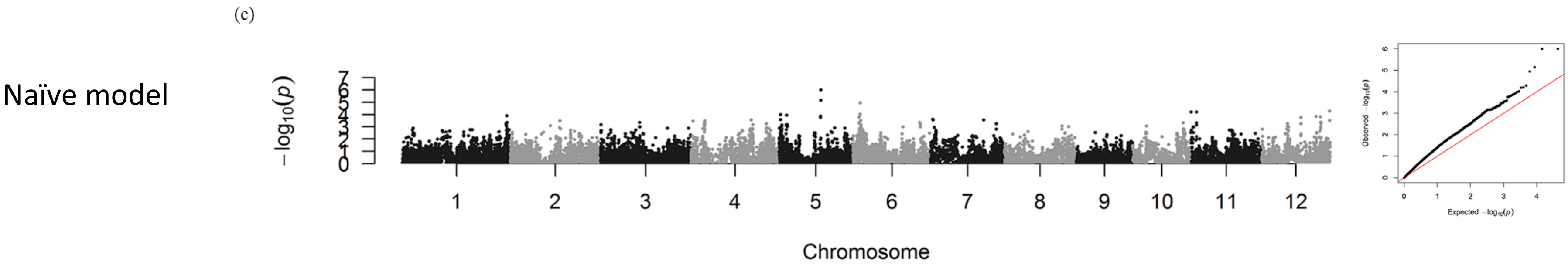

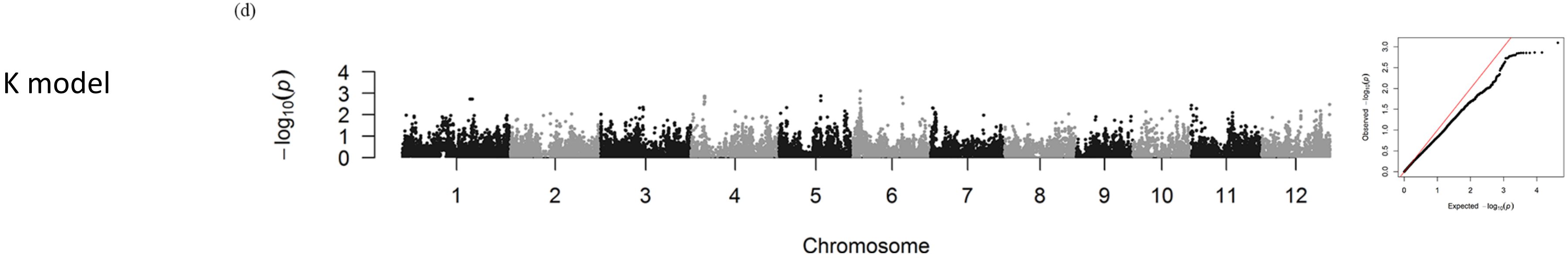

qL-whole panel

Naïve model

P model

K model

P+K model

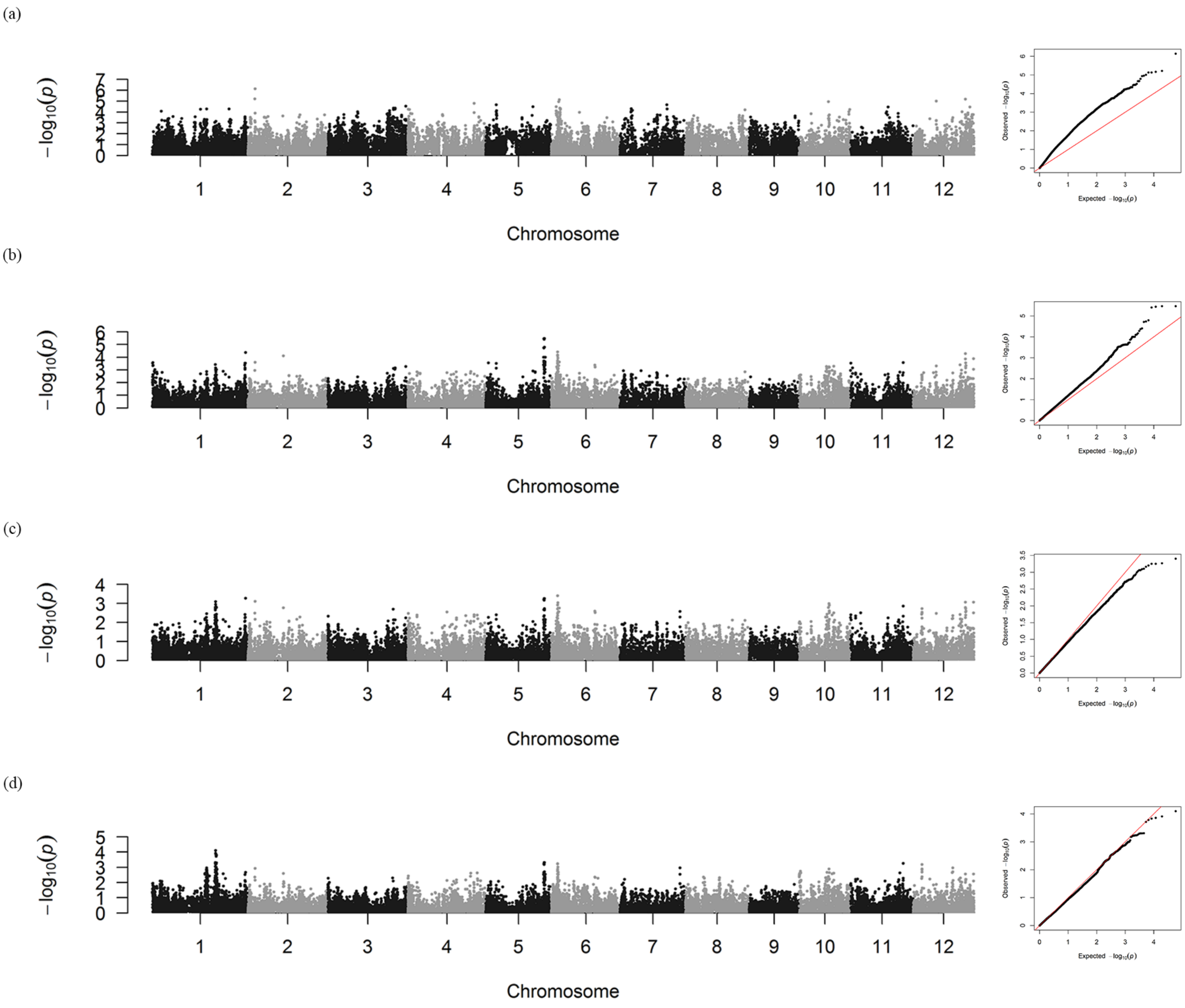

qL- *Japonica* panel

(a)

Naïve model

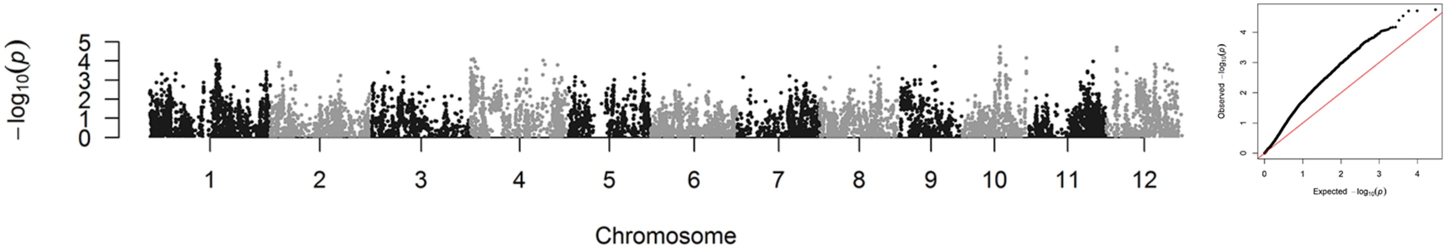

(b)

K model

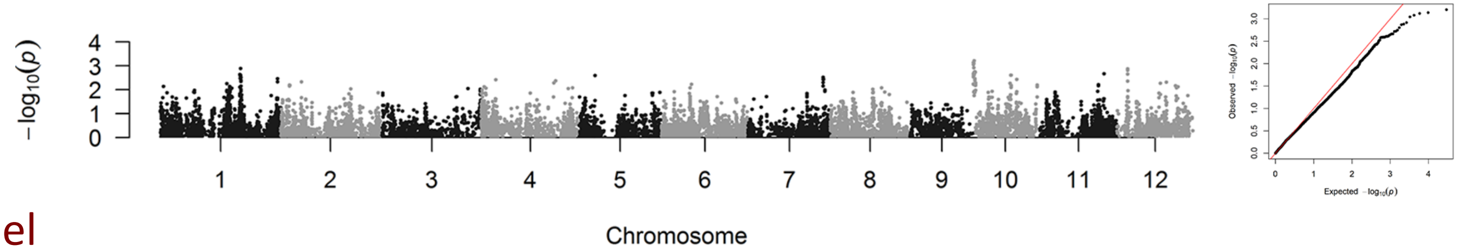

qL- *Indica* panel

(c)

Naïve model

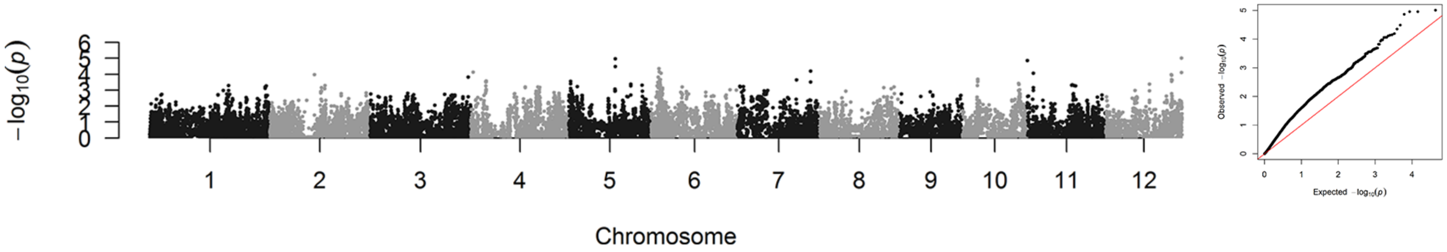

(d)

K model

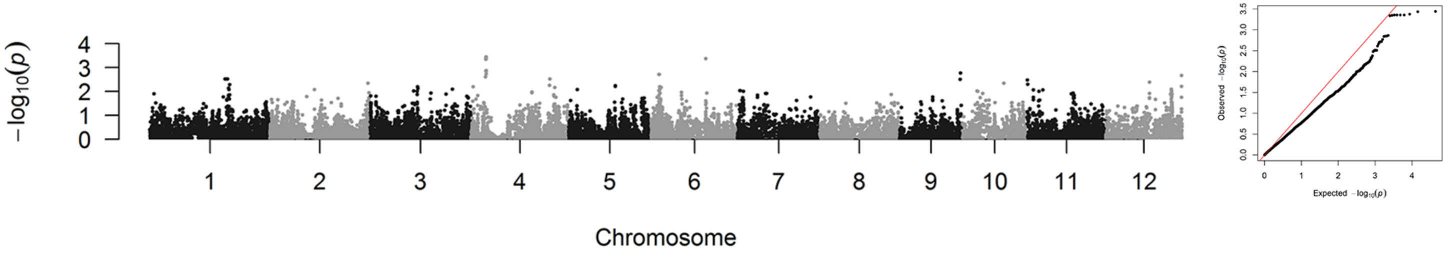

NPQ- whole panel

Naïve model

P model

K model

P+K model

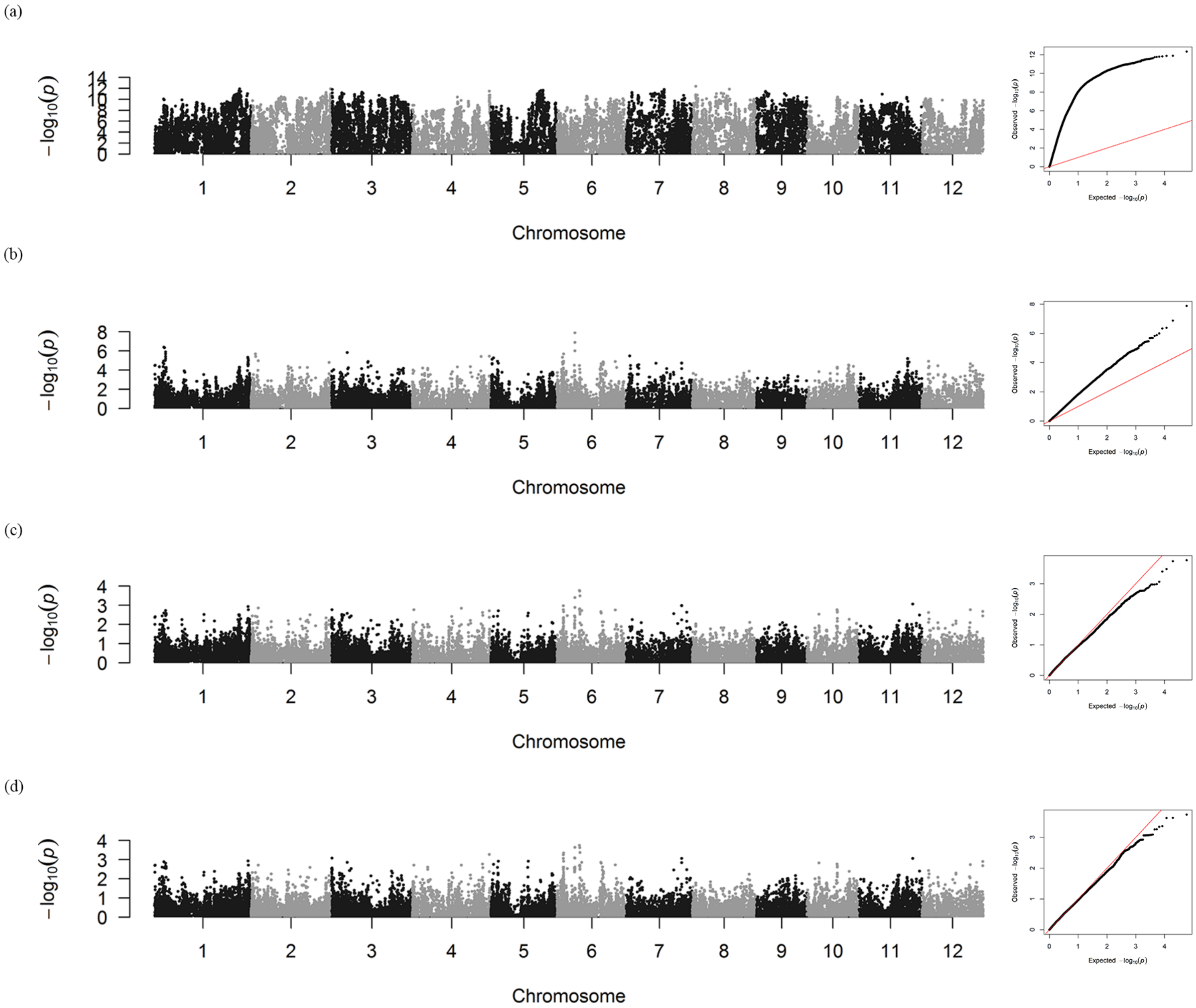

NPQ- *Japonica* panel

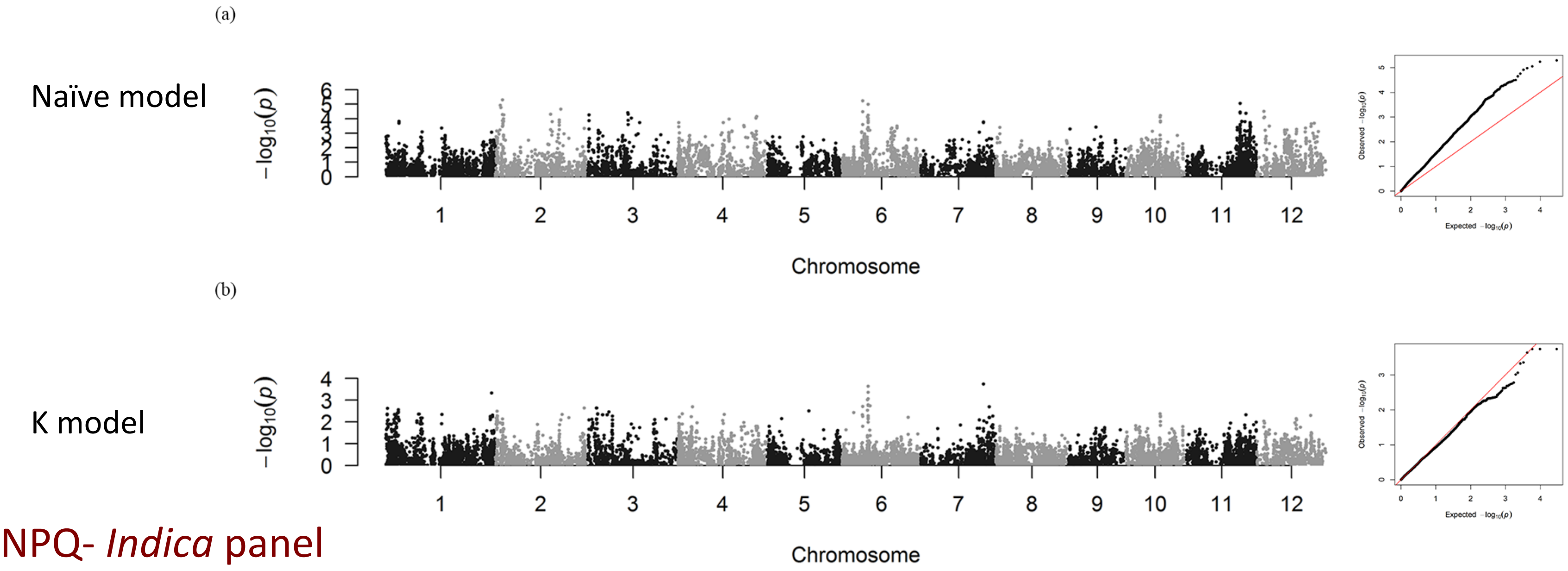

NPQ- *Indica* panel

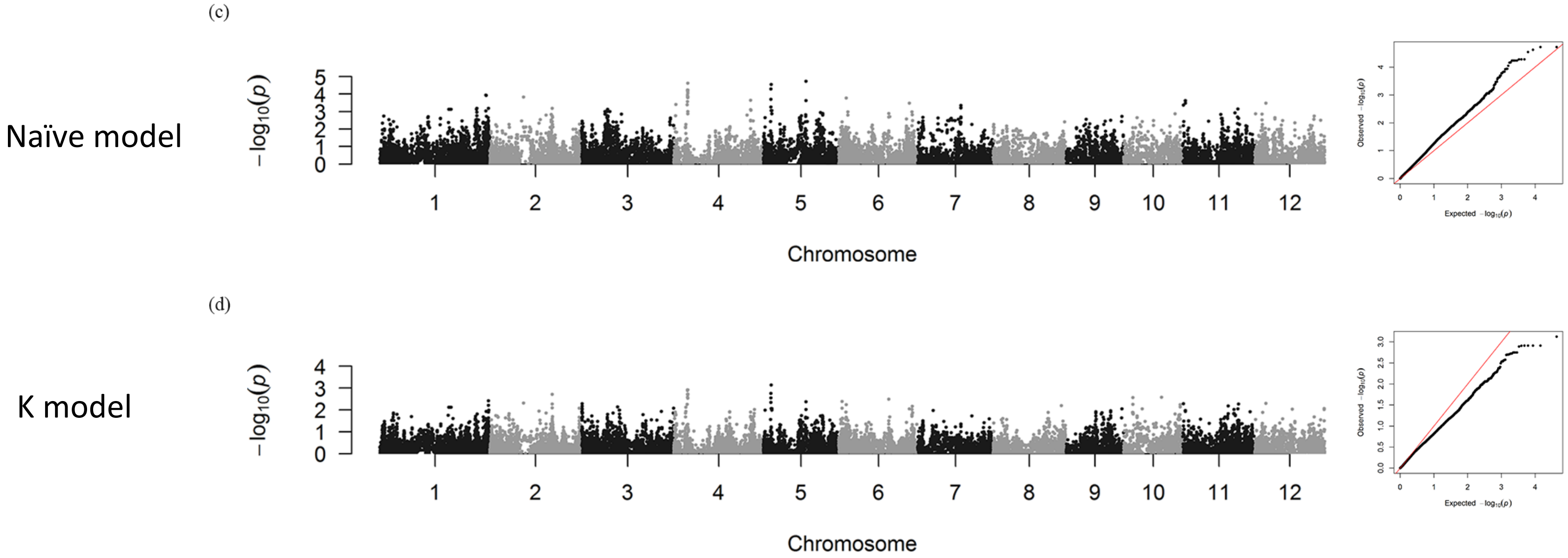

Supplement: Supplementary file 12 — Figure S6. Genome-wide association analysis of six chlorophyll fluorescence parameters in 232 diverse varieties or in two subspecies (Japonica and Indica). Manhattan plot showing the significance of each SNP tested by the appropriate statistical models described in “Methods”. (PDF 4610 kb) [file 12870_2019_1983_MOESM12_ESM.pdf]
